# Two-stage Non-Intrusive Load Monitoring method for multi-state loads

**DOI:** 10.1371/journal.pone.0312954

**Published:** 2025-01-08

**Authors:** Lei Wang, Xia Han, Yushu Cheng, Jiaqi Ma, Xuerui Zhang, Xiaoqing Han

**Affiliations:** 1 Taiyuan University of Technology, Taiyuan, China; 2 State Grid ShanXi Marketing Service Center, Taiyuan, China; Bahria University - Lahore Campus, PAKISTAN

## Abstract

The loads that have several working states cannot be accurately distinguished by the conventional Non-Intrusive Load Monitoring (NILM) methods. This paper proposed an improved NILM method based on the Resnet18 Convolutional Neural Network (CNN) and Support Vector Machine (SVM) algorithm to address the misidentification of multi-state appliances. The V-I trajectories of loads are at first classified with Resnet18. Then, load features with low redundancy is obtained through the Max-Relevance and Min-Redundancy (mRMR) feature selection algorithm from various operating states of loads that were not successfully classified. The SVM algorithm is developed for two-stage identification to achieve high accuracy of classification for identifying the multi-state appliances quickly. This proposed NILM method can significantly improve the accuracy of identification for multi-state loads. Finally, the Plaid dataset is acquired to validate the effectiveness and accuracy of the proposed method.

## 1. Introduction

With the construction of new type of power systems, optimizing dispatch [1] and coordinating supply-demand resources can promote the integration of renewable energy and contribute to achieving the goals of low-carbon and energy-saving [2]. Load monitoring is an important part of demand-side electricity management [3], and it can be categorized into intrusive and non-intrusive method [4]. Compared with the intrusive monitoring methods which has complicated installation, high equipment cost and difficult maintenance, NILM only requires to install an intelligent measuring device at the customer’s main feeder to collect information such as customer’s voltage and current. Due to low equipment cost and little impact on the daily life of the residents, NILM technology has become an important means of monitoring electricity consumption information [5].

NILM technology was proposed by professor Hart in 1992 [6]. With the rise of smart grids and smart homes in recent years, NILM research has attracted attention globally. Reference [7] proposed a load identification method based on deep learning and feature fusion for NILM technology, and analyzed the impact of different classification algorithms on load identification capabilities. Reference [8] optimized the network model structure based on the seq2point model, and the Time Convolutional Network (TCN) is employed to improve the performance of load decomposition. References [9, 10] adopted the steady-state features such as current waveforms, active power, reactive power, and transient waveforms for load decomposition. The proposed method improves the identification accuracy. Reference [11] took load recognition as an image classification task, and compares several transformation methods of Gramian angular field, Markov transition field, and recursive map. Reference [12] classifies load features into steady state features and transient features, and V-I trajectories in steady state features are adopted for load classification due to strong stability and high recognition. Reference [13] used RGB coding to fuse V-I trajectories with power information, instantaneous power and other features, which mapped more load information onto images and achieved accurate distinction of household loads. Reference [14] investigated and validated some hidden Markov model extension models and introduced some non-electrical characteristics such as the usage frequency of appliances and the correlation of each appliance to improve the decomposition accuracy.

Recently, research has focused on improving computation and energy efficiency of NILM. Reference [15] presents a weakly supervised approach to multi-label appliance classification based on a CRNN, and to exploit weakly labelled data. it reduces the quantity of strongly annotated data compared to supervised methods. Reference [16] proposes the scattering transform Non-Intrusive load monitoring (ST-NILM) that is a new integrated architecture based on the scattering transform (ST). This method requires less data and no need for data augmentation (DA) approaches. Reference [17] proposes a novel Convolutional transpose Reccurrent Neural Network (CtRNN) architecture focusing on reduced computational complexity and improving energy efficiency. Reference [18] proposes the few-shot Transfer learning (TL) based on meta-learning and relational network to improve the load recognition generalization performance, which does not require complex inference and recurrent structures. In addition, to effectively identify and differentiate low-power appliances, reference [19] presents a novel feature extraction approach that combines mono-fractal and multifractal analysis of appliance startup current transients. Reference [20] proposes a new mixed-integer linear programming (MILP) model which enables the identification of the operating state of each appliance and accurately fits the power consumption of each appliance.

Furthermore, some NILM research focuses on practical implementation and applications in the real world. Reference [21] presents NILM measurement method and application for real-time monitoring of electricity consumption by individual electrical energy consumers. Reference [22] presents NILM-Net for smart grid applications which can be queried on demand by grid participants. Reference [23] established a novel NILM system for Demand Response (DR) based on the DR requirement of hardware and software, following the practical load space and the explicit measuring criterion. In reference [24], an improved NILM method to identify electric vehicle battery charging in microgrid is presented. Reference [25] develops a smart home energy management system based on NILM for scheduling strategies of personalized appliances. In reference [26], a demand side personalized recommendation system by applying NILM method based on generalized particle filtering algorithms is proposed. Reference [27] developed harmonic analysis of motor current for sensor-less control and employed NILM for fault detection and diagnostics. The NILM has been used for appliance load monitoring in the interest of smart home and smart grid systems, more energy-related, safety-related and more intelligent services.

However, the appliances have several working states and the states of appliances are changing. Some intra-class and inter-class features of the multi-state appliances are similar. It can lead to the confusion of the identification results with other appliances. Reference [28] used the Alexnet CNN to classify the loads of the Plaid and whited datasets. However, only one state of the appliance is selected in the paper, and the identification of multi-state appliances have not been studied. Reference [29] investigated the typical working state of each appliance with the longest duration in the Plaid dataset. Most of studies above established feature libraries based on one single typical working state of the appliances, which lack comprehensive recognition and have shortcomings in identification accuracy. In recent years, some literatures have focused on the recognition of multi-state appliances. In reference [30], a few-shot learning (FSL) method based on voltage–current (V–I) trajectory signature has been proposed for different electrical appliances and the types of appliances constantly changing. However, multi-state appliances have not been involved. Reference [31] illustrates a NILM algorithm based on a convolutional neural network to handle multi-state appliances. However, it can recognize transition from one state to another. Reference [32] uses the K-means algorithm to solve the classification problem of multi-state loads. The current distortion features of loads are clustered and the Euclidean distance is used to weight the features. Reference [33] presents a load classification model based on SVM, which automatically adjusts the width of the Gaussian kernel according to different feature information to improve the classification performance. However, the identification accuracy of the multi-state appliances, such as water heaters and air conditioners, is low. Since the V-I trajectories of different working states of multi-state appliances are similar in shape to the trajectories of appliances with similar working principles, only one state of each appliance is not enough for identification accurately and can easily lead to confusion with the appliances with similar operating principles.

To address the low accuracy of identifying the multi-state appliances and similar load features for NILM algorithms, this paper proposes a two-stage load identification method for multi-state appliances based on the Resnet18 CCN and SVM algorithm. The cumulative sum control chart accumulation and event detection algorithm are utilized to capture the switching events of the appliance. The V-I trajectories at the switching moments are extracted, and load features with low redundancy is obtained through the mRMR feature selection algorithm. Then, two-stage load recognition process is presented. In the first stage, the Resnet18 network is employed to identify V-I trajectories, which can accurately identify most of the loads. In the second stage, for appliances that are not accurately identified in the first stage, the SVM algorithm is applied to recognize the combination feature for improving the identification accuracy. Finally, the Plaid dataset is acquired to validate the effectiveness and accuracy of the proposed method.

The contributions of the proposed NILM method include,

- Two-stage load identification method based on the Resnet18 convolutional neural network and SVM algorithm for multi-state appliances.
- The mRMR algorithm is developed to filter the types of load features, greatly reducing the redundancy of feature information and training computation.
- Features of each working state of appliances that are easy to be confused are built.
- This solution can significantly improve the accuracy of identification for multi-state loads that are easily confused.

## 2. Similar characteristics of V-I trajectories of multi-state loads

The V-I trajectories, as effective features for distinguishing loads, can intuitively reflect the impedance characteristics of household loads. However, the trajectories of electrical appliances with similar operating principles are also more similar, and the various states of multi-state appliances will affect the identification of other appliances.

**Fig 1:**
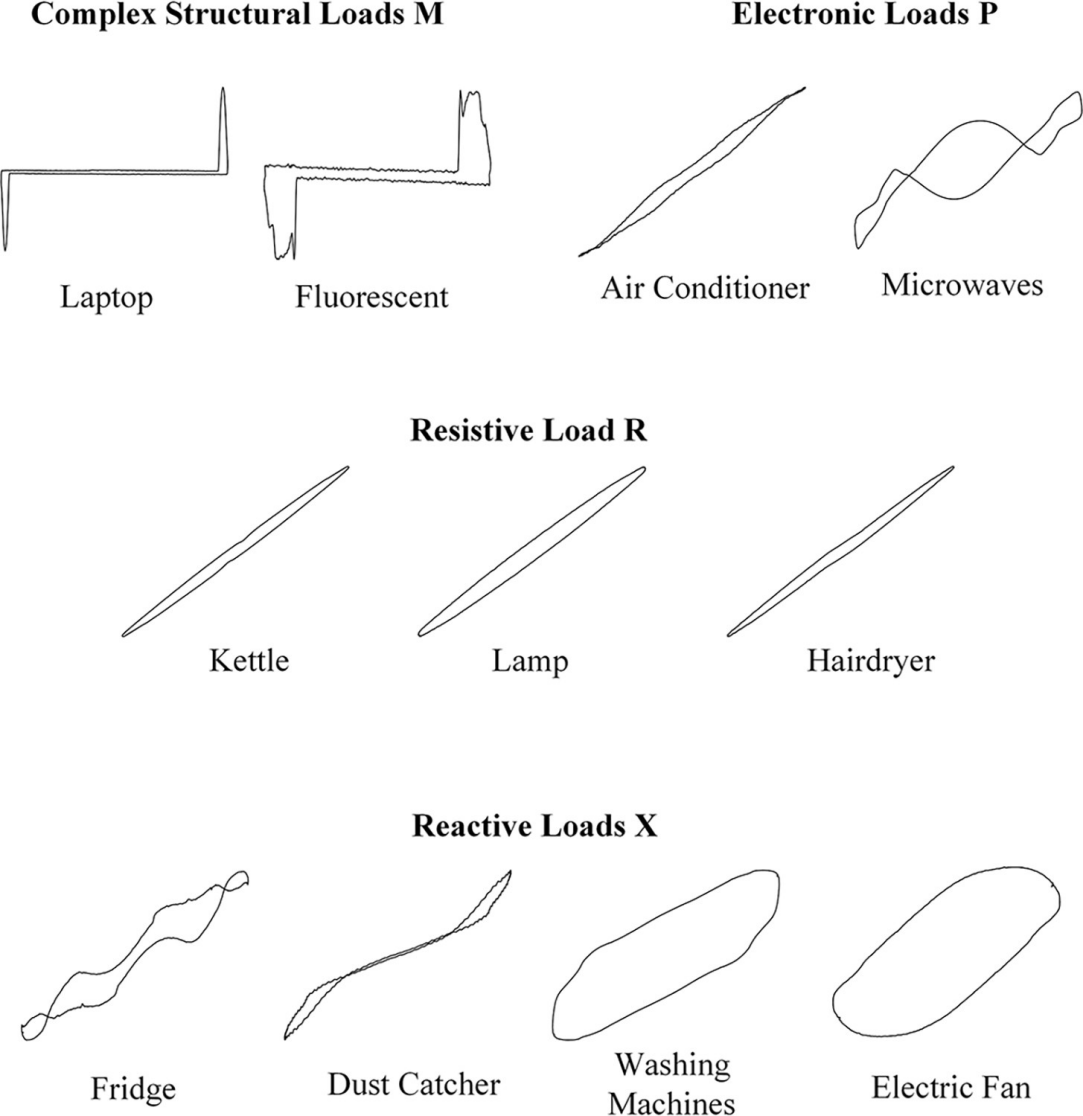
Classification of common household appliances and V-I trajectories.

Electrical appliances can be classified into seven types of loads based on their internal electronic components and circuit topology, including complex structure loads (M), power electronic load (P), resistive loads (R), reactive predominant loads (X), and others [34]. Complex structure loads (M) typically include high-power devices and multiple electrical systems, such as microwave oven. Power electronic loads (P) refers to electronic loads with Power Factor Correction (PFC), such as laptops. Resistive Loads (R) consist of a resistor connected to the front terminal with no phase angle shift between current and voltage, typical loads include water heaters, incandescent lamps, and hairdryers. Reactive loads (X) usually involve compressors, motors, or coolers, typical loads such as fans, washing machines, and refrigerators, etc. The above four categories contain most of the common household devices, and this paper focuses on the above four categories of electrical appliances, some of which are categorized and their V-I trajectories are shown in Fig 1.

In practical identification, not only is it challenging to distinguish appliances of the same type, but multi-state appliances are also easy to be confused. By extracting V-I trajectories of 21 working states of 11 common appliances, it is evident that the V-I trajectories of different states of multi-state appliances are different. However, some trajectories are highly similar with appliances of the same type.

To further verify the similarity between various trajectory shapes, the chi square distance [35] of histogram is used to calculate the similarity between V-I trajectory shapes of different appliances. This method reflects the probability distribution of pixel points in images and is suitable for V-I trajectory images without spatial hierarchy and texture structure. The V-I trajectory image is grayscaled and the probability distribution of the same pixel points is analyzed. The numerical range is [0,1], and the closer the value is to 0, the greater the similarity. The similarity comparison formula is as follows:

$$d(H_{1},H_{2})=\sum_{i}\frac{{\left(H_{1}(i)-H_{2}(i)\right)}^{2}}{H_{1}(i)}$$ (1)

where *d*(*H*_1_, *H*_2_) is the similarity, which compares the histograms of two images (H_1_ and H_2_). When H_1_ = H_2_, the two images are identical, and as numerator approaches 0, the deviation is smaller indicating greater similarity between the two images.

**Fig 2:**
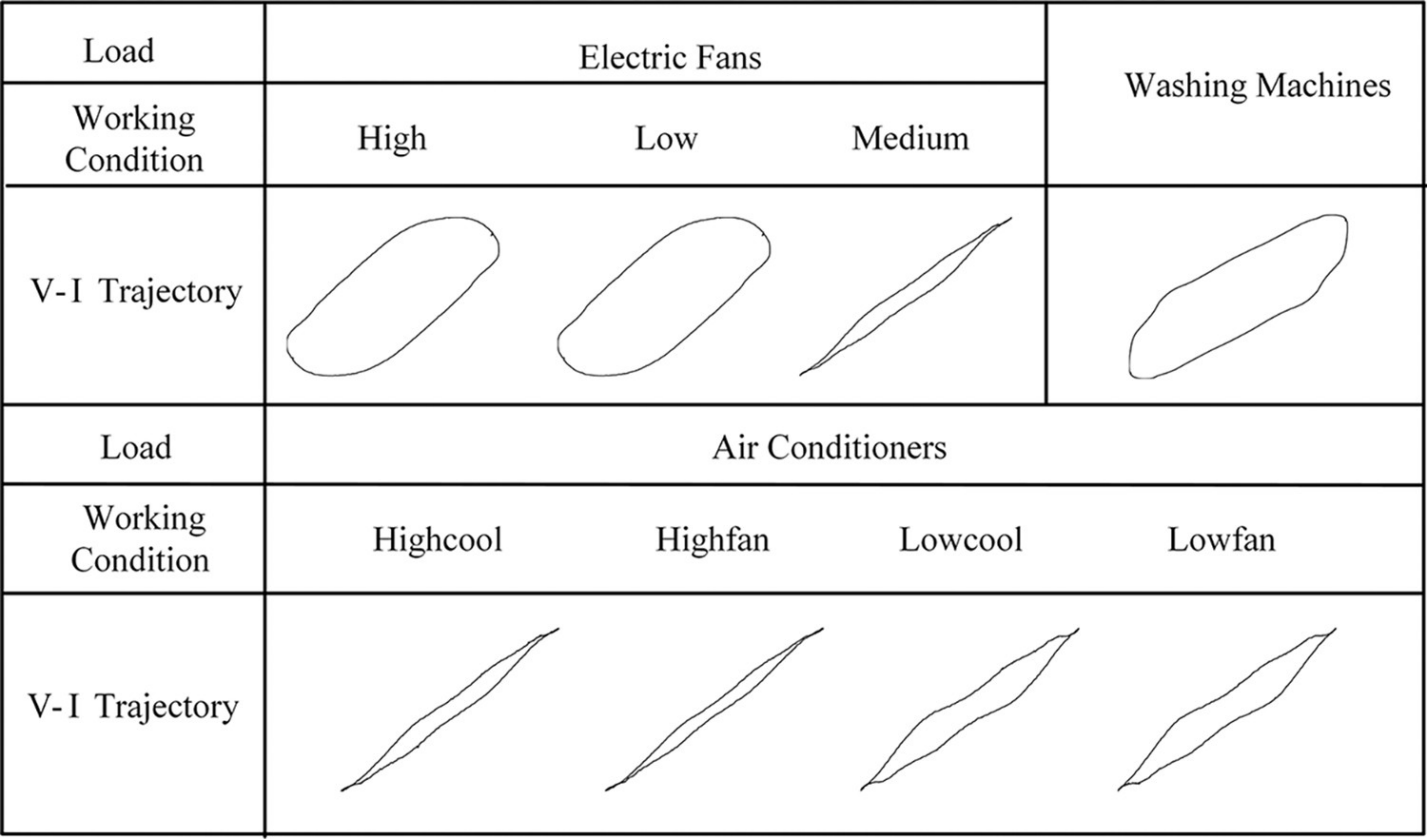
Comparison of V-I trajectories of states for three types of appliances.

Taking electric fan, washing machine, and air conditioner as examples, the V-I trajectories for each working state are obtained as shown in Fig 2, and the similarity results of each image are calculated using the chi square distance. In the case of the reactive load X, the electric fan has three working states, and its high-speed and low-speed trajectories are similar to washing machines, with similarities of 0.307 and 0.308, respectively. The medium-speed trajectory of the electric fan is also similar to the four working states of the air conditioner, with similarities of 0.252, 0.245, 0.297 and 0.294, respectively, which indicates relatively high similarity.

Similarly, the V-I trajectories of electric kettle, incandescent lamp and hairdryer in the resistive load R are extremely similar and the similarities are calculated to be as low as 0.223, 0.240 and 0.219, respectively.

It can be seen that the V-I trajectories are extremely similar for appliances with similar working principles. It is difficult to identify the appliances and eliminate the impact of multi-state appliances on identification with similar working principles based on only one single working state of each appliance. Therefore, it is necessary to model and extract more distinguishable features for each working state of multi-state appliances to improve identification accuracy.

## 3. Load identification based on resnet18-SVM

NILM collects voltage and current data by installed intelligent measuring devices at the customer’s mains to identify the type, operating status and power consumption of electrical devices on the customer’s side. And the results of the analysis can be used for planning electricity conservation by users to improve supply-demand balance in power grid.

Considering the various characteristics of appliances and the significant advantage of V-I trajectories in recognition, a two-stage recognition algorithm is adopted to enhance the accuracy of multi-state appliance recognition and reduce training computational complexity. This method employs the CNN model that has a greater advantage in recognizing the image and the SVM algorithm that is effective in recognizing feature vectors. In the second stage for feature selection, the mRMR feature selection algorithm is employed to filter out a group of features from the original feature set which have the highest relevance to the final output results and the smallest correlation between features to effectively reduce computational complexity.

**Fig 3:**
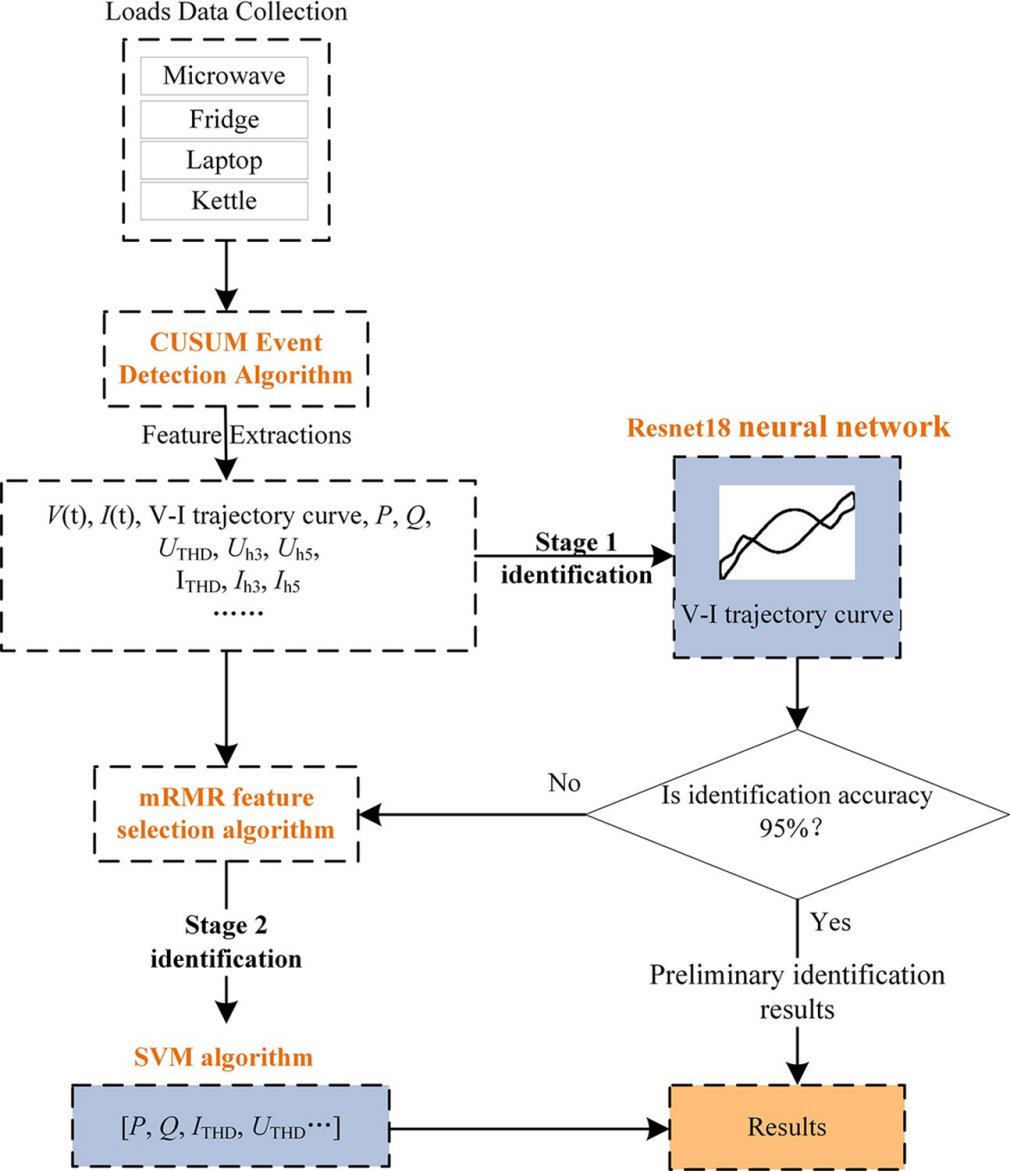
Diagram of load identification based on resnet18-SVM.

In the first stage, the Resnet18 model is used to recognize V-I trajectory images of each working state for various appliances, and the identification results with high accuracy is obtained in the paper. In the second stage, for multi-state appliances that are not accurately identified in the first phase and easily to be confused, the SVM algorithm is employed for further identification of the confused multi-state appliances. The two-stage load recognition model based on resnet18-SVM is proposed in this paper, and the overall flow is shown in Fig 3.

## 4. Phase I load identification

### 4.1. Construction of V-I trajectories

The V-I trajectories are graphical features constructed by extracting normalized voltage and current data during a steady state cycle, which is an important way to distinguish between different types of loads as follows [36].

1. Extract voltage $u=\{u_{1},u_{2},\ldots ,u_{k}\}$ and current $i=\{i_{1},i_{2},\ldots ,i_{k}\}$ within one stable cycle after a load switching event occurs, and normalize the voltage and current data to the range of [0,1]. The formula is as follows:
$$u_{nor}(t)=\frac{u}{\max(|u|)}$$ (2)
$$i_{nor}(t)=\frac{i}{\max(|i|)}$$ (3)
  where *u*_*nor*_(*t*) and *i*_*nor*_(*t*) represent the normalized voltage and current data at each moment, and max(|*u*|) and max(|*i*|) are the maximum values of voltage and current within one stable cycle.
2. When the resolution of the V-I trajectory image is set to N×N, create an N×N zero matrix ***N***, and map the normalized data into the zero matrix to generate the V-I trajectory matrix according to the following formulas.

$$u_{mn}=\lfloor u_{m}(t)\times (n-1)+1\rfloor$$ (4)

$$i_{mn}=\lfloor i_{m}(t)\times (n-1)+1\rfloor$$ (5)

$$N(u_{mn},i_{mn})=1$$ (6)

where *u*_*mn*_ and *i*_*mn*_ are the row and column indices of the zero matrix N, and ⌊ ⌋ represents the floor function. The resulting image, after normalization and high-resolution pixelation, is a 25x25 matrix, where white indicates matrix elements with a value of 0, and black indicates matrix elements of 1.

The V-I trajectory curves of each working state of the 11 appliance classes in the Plaid dataset are constructed based on this method. The feature trajectories extracted in the first phase of recognition are used to establish a feature library.

### 4.2. CNN structure design

Since CNN can deeply explore the deep feature information in images, the Resnet18 model, proposed in 2015, is a typical CNN model due to outstanding performance in image recognition and classification tasks. Resnet18 means residual network with 18 layers and it belongs to the ResNet family of networks, which is introduced by researchers to address the issue of vanishing gradients in neural networks. It can enable the training of networks with hundreds or even thousands of layers. By using inter-layer residual connections, multiple Resnet blocks are established to prevent subsequent convolutional layers from losing original feature elements. In the first phase of this paper, the Resnet18 model is employed, and V-I trajectory curves are taken as image inputs for preliminary classification of different types of devices.

**Fig 4:**
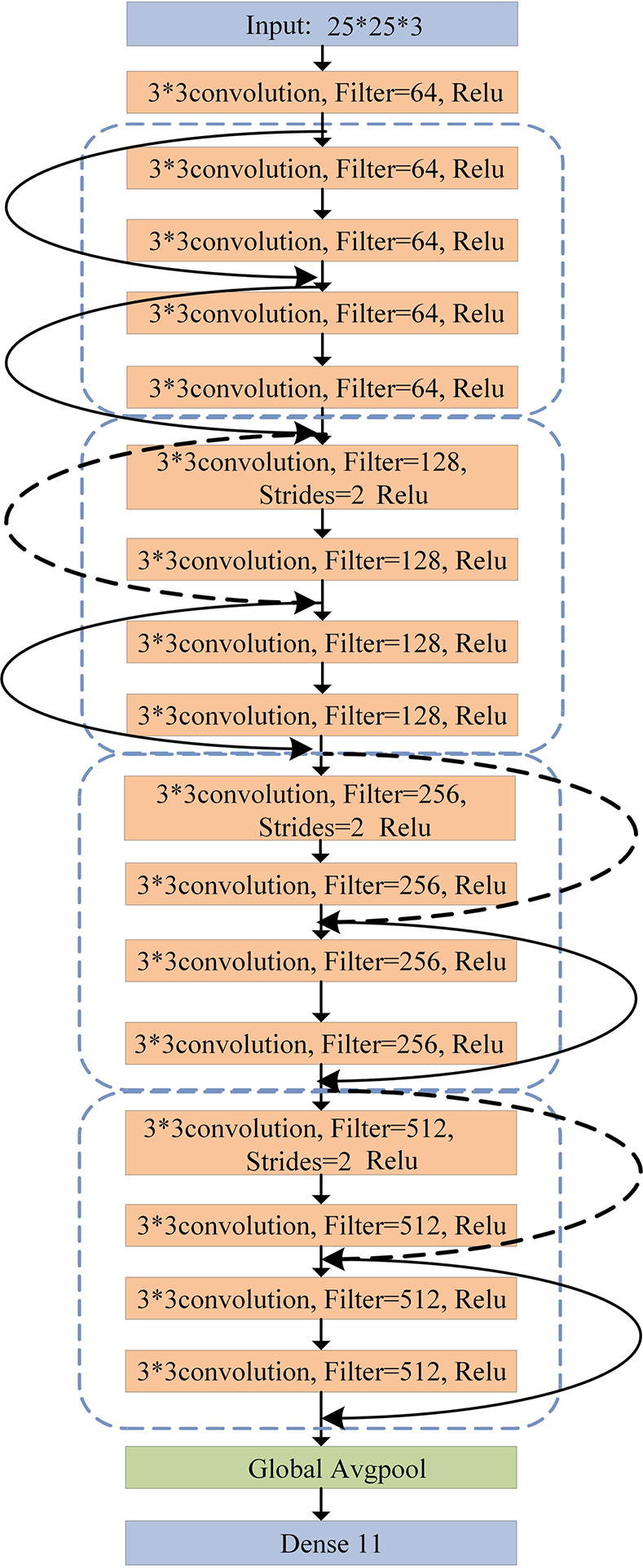
Resnet18 model structure.

The structure of Resnet18 model used in this paper is shown in Fig 4. The original Resnet18 model cannot be directly applied to the classification of the Plaid dataset. Therefore, the model is modified by improving its parameters, which is adapted to the requirements of load identification. The input image passes through a convolutional layer and enters 8 Resnet blocks, which consisting of two convolutional layers. Finally, a fully connected layer is used for classification, and the number of vectors generated is equal to the number of appliance categories to be recognized 11. The model is trained for 800 epochs with a learning rate of 0.0001.

## 5. Phase II load identification

### 5.1. mRMR algorithm for feature selection

The representative features that contains the largest amount of information and the smallest redundancy for load recognition can effectively improve recognition accuracy, enhance robustness of the algorithm, and accelerate the calculation speed.

The mRMR algorithm calculates the maximum correlation and the minimum redundancy among the features, thereby finding a set of features in the original feature set with the maximum correlation to the final output result and the minimum correlation between features. The goal of mRMR is to find a set of features that are highly relevant to the class while being as non-redundant as possible.

1) The probability density functions *p*(*x*), *p*(*y*) and *p*(*x*,*y*) are defined. The value of mutual information is given by:

$$I(x,y)=\iint p(x,y)\log\frac{p(x,y)}{p(x)p(y)}dxdy$$ (7)

2) The maximum correlation *D*(*S*,*c*) and minimum redundancy *R*(*S*) is derived as:

$$D(S,c)=\frac{1}{\left|S\right|}\sum_{x_{i}\in S}I(f_{i};c)$$ (8)

$$R(S)=\frac{1}{{\left|S\right|}^{2}}\sum_{f_{i},f_{j}\in S}I(f_{i};f_{j})$$ (9)

where *f*_*i*_ represents the *i*^th^ feature variable, *c* is the class variable, and *S* is the feature set.

3) Importance indicators is as:

ϕ=max[D(S,c)−R(S)] (10)

The features can be categorized into steady-state features and transient features according to the different operating states of the loads. The steady-state features are calculated based on the sampling points of one cycle at steady state. The transient features are calculated with sampling points during transient processes. Although transient features contain a lot of information, they are not stable enough. Therefore, the transient features are limited and used relatively less.

The loads features that commonly are used for load identification have: active power (P), reactive power (Q), total harmonic distortion of current, the 3^rd^, 5^th^, 7^th^, 9^th^, 11^th^ harmonic current (*I*_h3_, *I*_h5_, *I*_h7_, *I*_h9_, *I*_h11_), total harmonic distortion of voltage, the 3^rd^, 5^th^, 7^th^, 9^th^, 11^th^ harmonic voltage (*U*_h3_, *U*_h5_, *U*_h7_, *U*_h9_, *U*_h11_), root mean square (RMS) of current, current span, V-I trajectory area, V-I trajectory symmetry, V-I trajectory direction, Y-axis positive intercept of V-I, Y-axis negative intercept, and so on.

**Fig 5:**
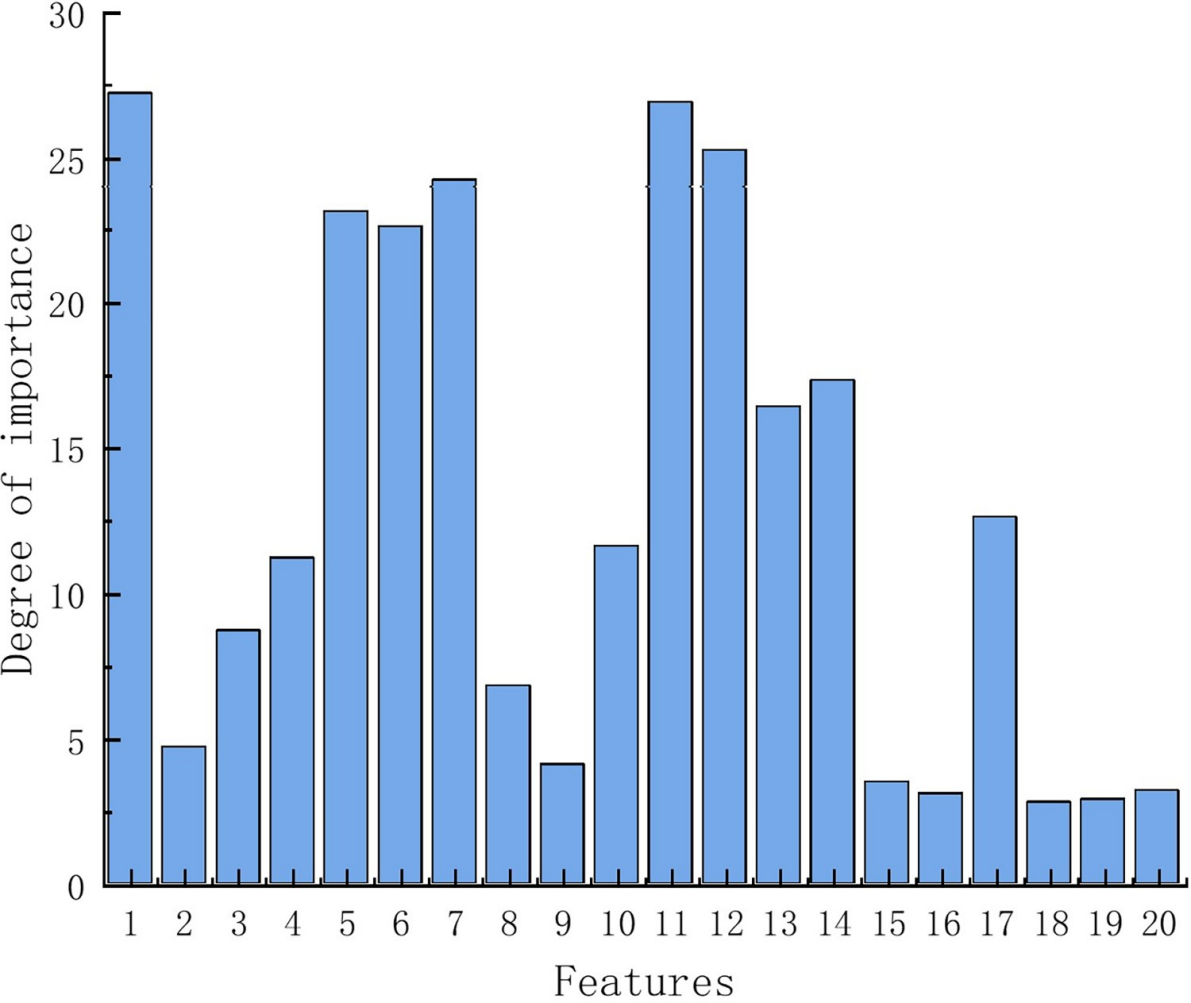
Importance ranking of feature.

The importance indicators *Ф* of above features are calculated based on the mRMR method and the ranking of these features is obtained in the order as shown in Fig 5. A higher importance value indicates better inter-class distinctiveness and lower correlation among features. The features ranked in the top i can be selected to form the optimal feature combination.

As seen in Fig 5, there is a significant difference. In this paper, the top six features in the ranking are selected for the second stage of identification based on the importance indicators *Ф*, which are *P*, *U*_h5_, *U*_h7_, *I*_h5_, *I*_h7_, and *I*_h9_.

### 5.2. SVM algorithm based feature classifier

The SVM that is the supervised learning algorithm has excellent performance for classification with small samples, nonlinear and high-dimension [37]. SVM aims to find a hyperplane that best separates the data points of different classes. This hyperplane is chosen in such a way that the margin between the two classes is maximized, which helps in achieving better generalization for unseen data. In order to be applied to the multi-class scenario, it is necessary to construct the suitable multi-classifiers for SVM. In this paper, classifiers are constructed based on the indirect method which includes one-versus-all (OVA) and one-versus-one (OVO). Compared with the OVO method, the OVR method requires fewer constructed classifiers and has faster training speed. Considering the variety of work states in the second stage algorithm, the OVA method is adopted to construct classifiers. In order to improve the training speed and recognition accuracy of the algorithm, reduce the training time, and improve the recognition efficiency, a total of 14 classifiers need to be constructed for recognizing 14 operating states.

## 6. Case analysis

The hardware and software platform for the load recognition experiments in the paper is established. The hardware consists of an Intel Core i5-12700H @2.50 GHz, 16GB DDR4 memory, and a GeForce GTX 1050Ti (4GB VRAM) 64-bit Windows system computer. The software platform is based on the open-source Python 3.8 with TensorFlow 2.0 for implementing deep neural network modeling. In order to validate the algorithm’s effectiveness, the Plaid public dataset is chosen as the experimental data.

The Plaid dataset is a widely utilized, high-frequency sampling, publicly available dataset in the field of NILM research. The Plaid dataset contains sample data from 55 households in Pennsylvania, USA, covering 11 different appliances. The voltage and current data are recorded with 30 kHz sampling frequency for each running device for over 2 seconds. The Plaid dataset has different brands and operation states for similar loads, which have high intra-class differences. Using V-I trajectories as load classification identifiers, it is verified that the good recognition results are achieved when the sampling frequency is greater than 4 kHz [38]. The Plaid dataset with 30 kHz sampling frequency meets the experimental requirements. The organized load categories and their corresponding numbers in the Plaid dataset used in the paper are presented in Table 1 below.

**Table 1:** Plaid dataset.

Load	Data records	Number of states
air conditioner	66	5
fluorescent	175	1
electric fan	115	4
fridge	38	2
hairdryer	156	10
kettles	35	1
incandescent lamp	114	1
laptop	172	1
microwaves	139	4
dust catcher	38	1
washing machines	26	1

### 6.1. First stage identification

In this paper, the V-I trajectory images of loads are used as input, and the Resnet18 algorithm is employed for preliminary load classification. Although some appliances are different brands and models, they have high similar V-I trajectories. Therefore, different brands of appliances are randomly selected for testing. In the first stage, the operating state with the longest duration is selected for each appliance as a typical working state to extract the trajectory, and thus the number of neurons in the last fully-connected layer of the Resnet18 algorithm is set to 11. According to the Resnet18 model, the model is trained with the divided training set. The feature data for all appliances are grouped into a training set and a test set according to 7:3.

The confusion matrix, F1 score and accuracy are adopted to evaluate the algorithm in the paper. The diagonal number in the confusion matrix represents the number of the correctly identified samples, while the non-diagonal number represents the number of the misidentified samples.

The F1 score reflects the performance of the algorithm, and it is the harmonic mean of the precision *P*_*re*_ and the recall *R*_*re*_. The accuracy is the ratio of correctly identified samples to the total number of samples in the test set. The formulas are as follows:

$$P_{re}=\frac{T_{P}}{T_{P}+F_{P}}$$ (11)

$$R_{re}=\frac{T_{P}}{T_{P}+F_{N}}$$ (12)

$$F_{score}=\frac{2P_{re}R_{re}}{P_{re}+R_{re}}$$ (13)

$$A_{acc}=\frac{a}{A}$$ (14)

where *T*_*P*_ is the true positive which represents the number of samples that the true class is positive and the predicted class is also positive, *F*_*P*_ is the false positive which represents the number of samples that the true class is negative and the predicted class is positive, *F*_*N*_ is the false negative which represents the number of samples that the true class is positive and the predicted class is negative, *a* is the number of samples that are correctly classified, and A is the total number of samples in the test set.

**Fig 6:**
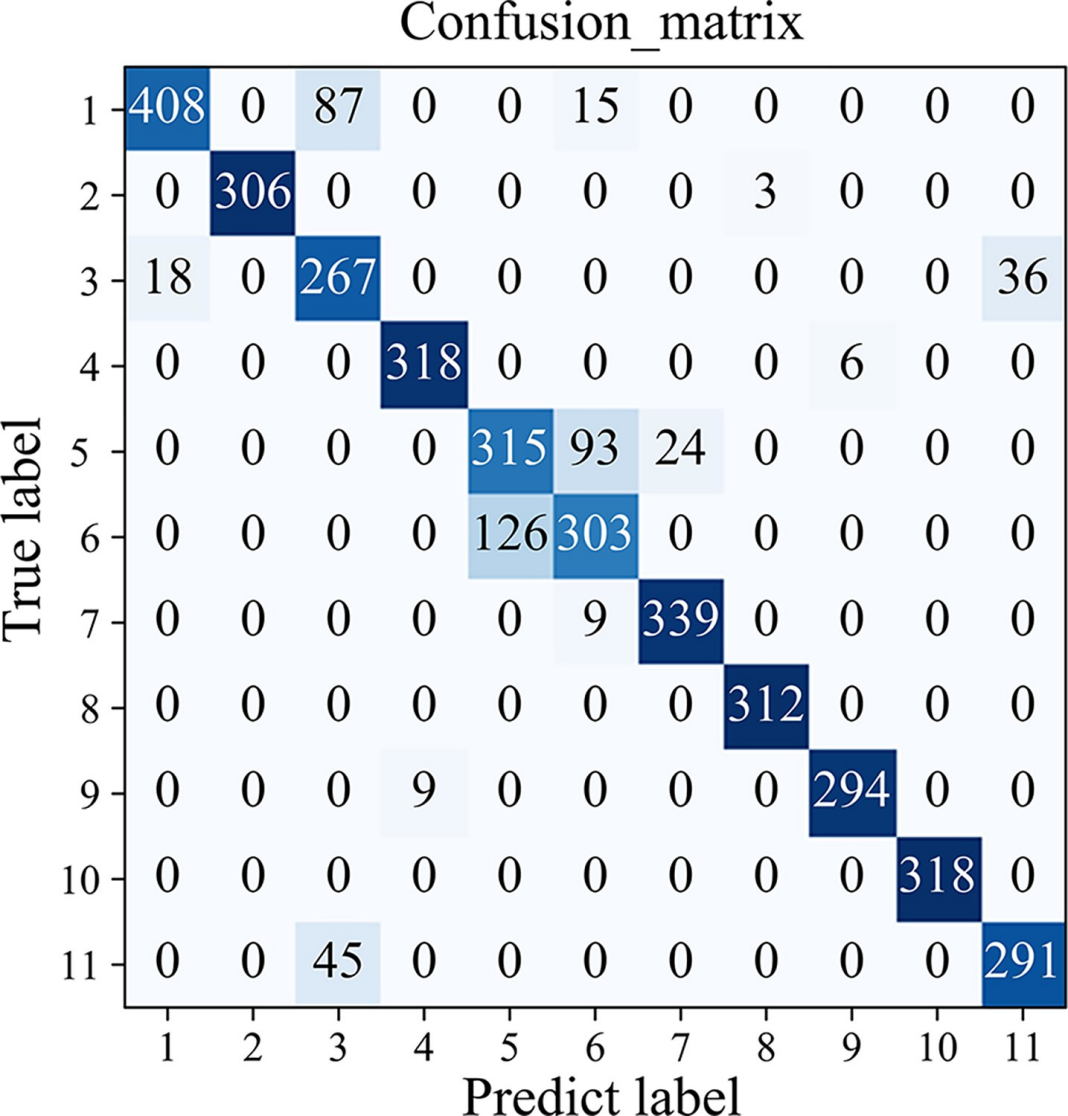
Stage 1 confusion matrix.

The accuracy of the validation set has a large gap for different appliances after the first step of recognition using Resnet18, ranging from 85% to 99%. The output results are presented in a confusion matrix in Fig 6, and the recognition accuracy for each appliance is shown in Table 2.

**Table 2:** Recognition accuracy of each appliance(%).

Label	Load	Acc (%)	Label	Load	Acc (%)
1	air conditioner	80	7	incandescent lamp	95
2	fluorescent	99	8	laptop	100
3	electric fan	83	9	microwaves	97
4	fridge	98	10	dust catcher	100
5	hairdryer	73	11	washing machines	86
6	kettles	70	Total		89.5

It can be observed from Fig 6 that after the identification in the first stage, appliances 1 (air conditioner), 3 (electric fan), 5 (hairdryer), 6 (electric kettle), and 11 (washing machine) are prone to be misclassified as other types of appliances, with accuracy of 80%, 83%, 73%, 70%, and 86%, respectively. It can be seen that air conditioner, electric fan, and washing machine are easy to be confused for recognition. Both air conditioners and electric fans are multi-state appliances, and the V-I trajectory features of different states vary greatly, making it easy for them to overlap with trajectories of other appliances with similar structures and leading to confusion. The Highcool and Highfan states of the air conditioner have a great similarity to the Medium state of the electric fan, while the High and Low states of the electric fan have a great similarity to the washing machine’s trajectory, as shown in Fig 2. Therefore, it is prone to misjudgments for recognition. Although the hairdryer and electric kettle are both multi-state appliances, the V-I trajectories for each state are essentially the same. They are the heating-type appliances and can be categorized as resistive loads (R) with similar internal constructions. Thus, it is highly susceptible to be misidentified, and additional features are required to distinguish them.

The recognition accuracy of appliances in classes 2, 4, 7, 8, 9 and 10 is close to 99%, and the identification results are considered to be accurate. In the second stage, further recognition is performed for the five types of appliances that fail to be accurately classified in the first stage. The multi-dimensional features of each working state are studied to improve the accuracy of model recognition.

### 6.2. Second stage identification

The feature vectors of 14 working states of five types of appliances which were not accurately identified in the first stage are extracted based on the above method. These feature vectors are used as inputs to the SVM classifier in the second stage of recognition which has higher capability of differentiation. The SVM classifier is constructed based on the OVA method and 14 classifiers are created for training. The feature data for all appliances are grouped into training set and test set according to 7:3 ratio.

Each label corresponding to the working state of the appliance is described as follows: the tens digit corresponds to the load label represented in the first stage, the units digit increases from 1 that represents different working state categories for multi-state appliances. For example, 11 represents the Highcool working state of the air conditioner with a label of 1. The special label ‘00’ represents the washing machine in the second stage with a label of 11. The results in the second-stage identification are the tens digit corresponding to the appliances, whose label is consistent with the first stage. The confusion matrix combines different states of the same type of appliance into one category.

**Fig 7:**
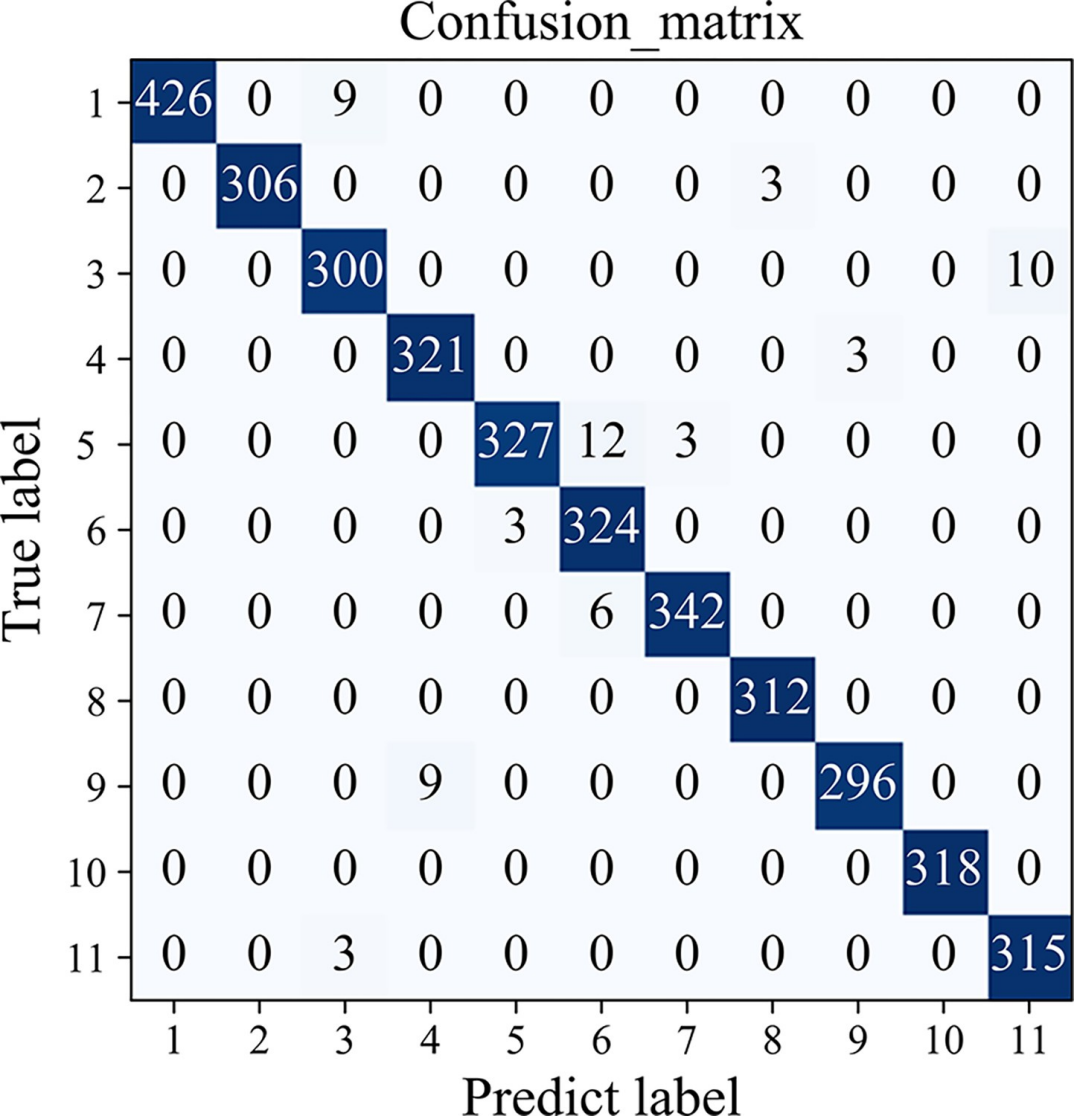
Stage 2 confusion matrix.

After the second-stage identification, a detailed confusion matrix for multi-state appliances is obtained, as shown in Fig 7. It can be seen from this confusion matrix that the accuracy of appliance recognition is significantly improved. Especially for several types of multi-state appliances with similar V-I trajectories, the accuracy is improved to 98%, 96%, 95.6%, 99%, and 99%, respectively. The recognition accuracy for each appliance is shown in Table 3.

**Table 3:** Recognition accuracy of each appliance(%).

Label	Load	Acc (%)	Label	Load	Acc (%)
1	air conditioner	97.9	7	incandescent lamp	98
2	fluorescent	99	8	laptop	100
3	electric fan	96	9	microwaves	97
4	fridge	99	10	dust catcher	100
5	hairdryer	95.6	11	washing machines	99
6	kettles	99	Total		98.6

**Fig 8:**
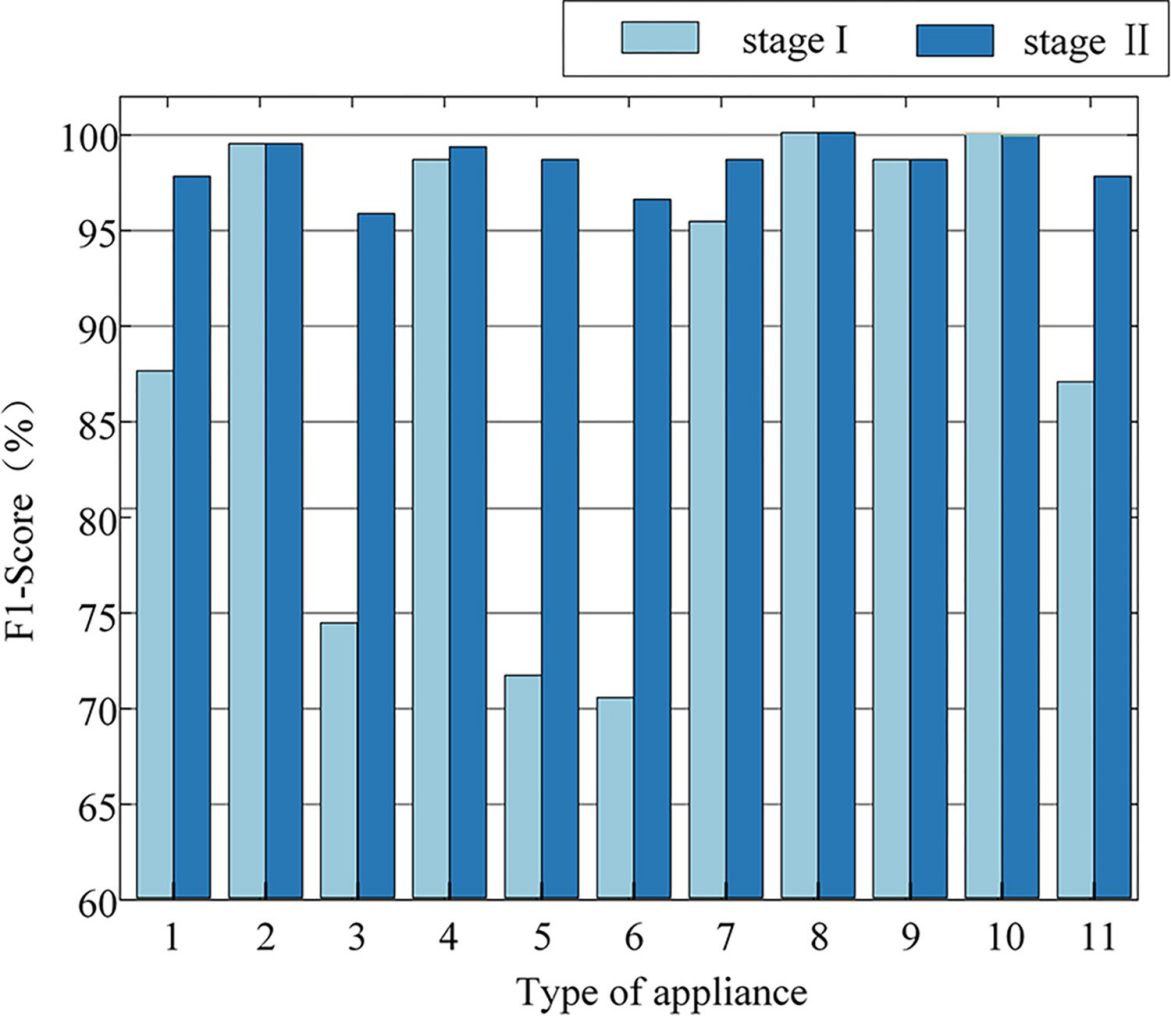
Comparison of F1-score (%) for the two phases of each load.

The F1 scores for each type of appliance which is calculated from the confusion matrix are used to analyze the recognition capability of algorithms for each class of appliances after every stage of recognition. The F1 scores of each stage of recognition are shown in Fig 8. It can be obviously seen that the recognition effectiveness of each load after the second stage is significantly improved and the F1 score increases to 98.22%. Especially for the easily confused classes 1, 3, 5, 6, and 11 appliances in the first stage, the recognition improvement is more obvious.

In conclusion, even though the V-I trajectory features of these five types of loads are similar, the second-stage SVM classifier for further training and classification, having additional physical information for assistance in identification, greatly enhances the uniqueness of load features. Therefore, the two-stage collaborative recognition can achieve better classification results.

### 6.3. Analysis and discussion

In order to further validate the effectiveness of the algorithm proposed in this paper, the different neural networks and classification models are used for comparison and analysis with the same dataset, and the comparison results are shown in Table 4. These models and their configurations are presented in the S1 Appendix.

**Table 4:** Comparison results with different algorithms.

Characteristics	Identification Models	Acc (%)
V-I Trajectory	VGG16	89.2
	Resnet18	89.5
Power (Output)	K-Means	70.7
	SVM	73.2
Current Harmonic RMS	K-Means	75.1
	SVM	76.2
Six-Dimensional Eigenvector (Single-State)	K-Means	83.6
	SVM	89.3
V-I Trajectory+Single-State Eigenvectors	VGG16+SVM	93.6
	Resnet18+SVM	95.8
V-I Trajectory+Multi-State Eigenvectors	VGG16+SVM	96.3
	Resnet18+SVM	98.6

The recognition accuracy reaches only 89.5% when using the Resnet18 network to recognize the V-I trajectory feature of individual appliances. Especially for multi-state appliances with similar internal structures that V-I trajectories are confused seriously, the recognition results are not satisfied. Although the SVM classifier is more effective than K-Means method in recognizing multi-dimensional feature vectors, the accuracy is still only about 89.3%. The accuracy is improved by combining the two methods into a two-stage recognition algorithm. However, there are still misjudgements in the identification of some multi-state appliances, such as the electric kettle and hairdryer.

The first stage utilizes a Resnet18 convolutional neural network to train and test on the U-I trajectory images of loads, with an image size of only 28*28 pixels. The average training duration for this stage is approximately 6 minutes, while the average testing duration requires only 1 second. The second stage uses an SVM algorithm to identify multi-dimensional vectors composed of load characteristic data. By applying the mRMR feature selection algorithm to rank the importance of multiple load features and selecting six key features, the computational complexity and time of the algorithm is significantly reduced, enhancing recognition efficiency. The average training duration for the second stage is about 2 minutes, and the average testing duration is 1 second. Although an extra stage in the recognition process is added, algorithm is optimized for recognition steps, such as reducing redundant calculations and employing more efficient data preprocessing strategies. The amount of computation and overall time does not increase much. Additionally, it enhances the recognition effect for multi-state loads and improves recognition accuracy. Therefore, this proposed method achieves a good balance between computation and accuracy, providing a more feasible option for practical applications.

It can be seen that the two-stage load recognition method proposed in this paper presents a higher accuracy with the same dataset. The number of misclassifications significantly reduces for the electric kettle and hairdryer. The electrical characteristics of different working states of appliances are incorporated to assist identification, which can contain more dimensions of information about the load, and the excellent performance of identification can be achieved especially for appliances with the similar internal structures.

## 7. Conclusion

In this paper, a two-stage non-intrusive load identification algorithm based on load multi-state features is proposed to address the problem of low recognition accuracy of multi-state appliances. The monitoring data from Plaid dataset is applied to verify the proposed algorithm and the following conclusions are obtained.

1) The accuracy of the proposed method is improved by combining the two methods into a two-stage recognition algorithm with Resnet18 and SVM. The recognition accuracy reaches 98.6%. Compared with the single stage method, the proposed method has a great improvement. Meanwhile, the proposed method also has better performance of identification, compared with VGG16+SVM two-stage method.

2) The mRMR feature algorithm is used to filter the types of load features, which greatly reduces the redundancy of load feature information and the training computation.

3) In the second stage of identification, features are extracted separately for each working state of appliances that are easy to be confused in the first stage. This greatly improves the identification accuracy of algorithm for multi-state appliances with similar characteristics. It is a satisfied solution to the misidentification of multi-state appliances that are easily confused. The proposed method has significantly improved the identification accuracy for multi-state loads.

4) This paper primarily focuses on load identification of household appliances. It has not involved the recognition of equipment in large enterprises and factories. Nevertheless, large enterprises and factories play the important roles in demand-side management. Therefore, load identification based on the NILM for industrial loads is attractive and should be studied in future.

## Supporting information

S1 Data Processing results and code. (ZIP)

S1 Appendix Summary table of different models and their configurations employed in the paper. (DOCX)
