# Supplementary figures and images for: Two-stage Non-Intrusive Load Monitoring method for multi-state loads
Source: PLoS One. 2025 Jan 8;20(1):e0312954. doi: 10.1371/journal.pone.0312954 (PMC11709270; doi:10.1371/journal.pone.0312954)

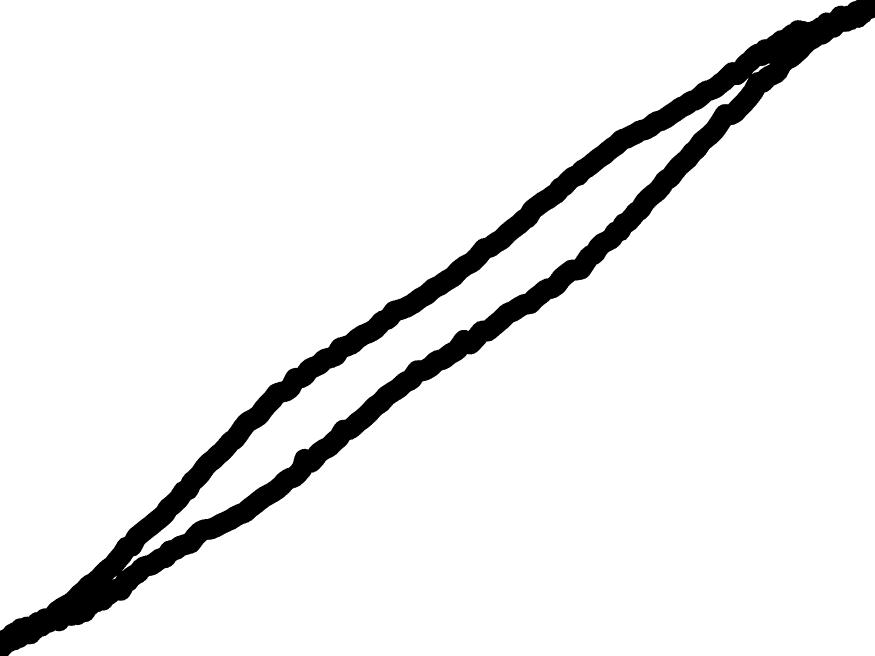

Supplement: S1 Data — (ZIP) [file pone.0312954.s001.zip › S1 data/1.V-I trajectories/0/1 highcool_1057/100_0.jpg]

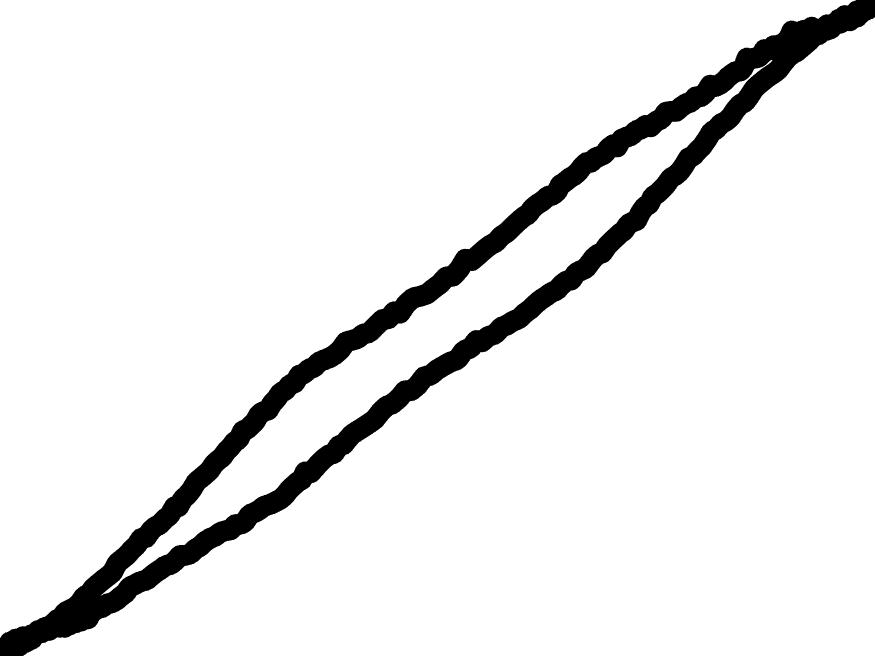

Supplement: S1 Data — (ZIP) [file pone.0312954.s001.zip › S1 data/1.V-I trajectories/0/1 highcool_1057/101_0.jpg]

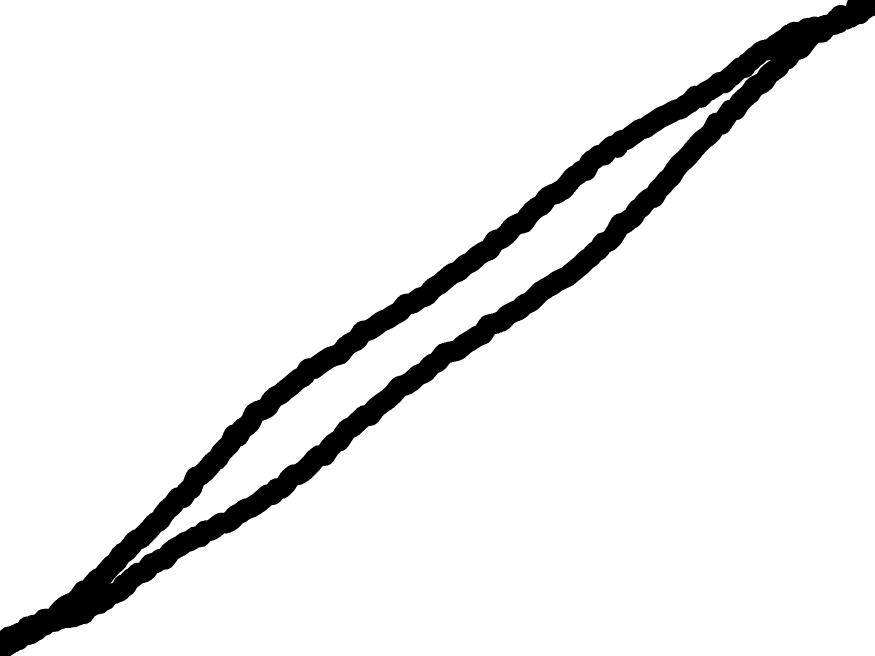

Supplement: S1 Data — (ZIP) [file pone.0312954.s001.zip › S1 data/1.V-I trajectories/0/1 highcool_1057/105_0.jpg]

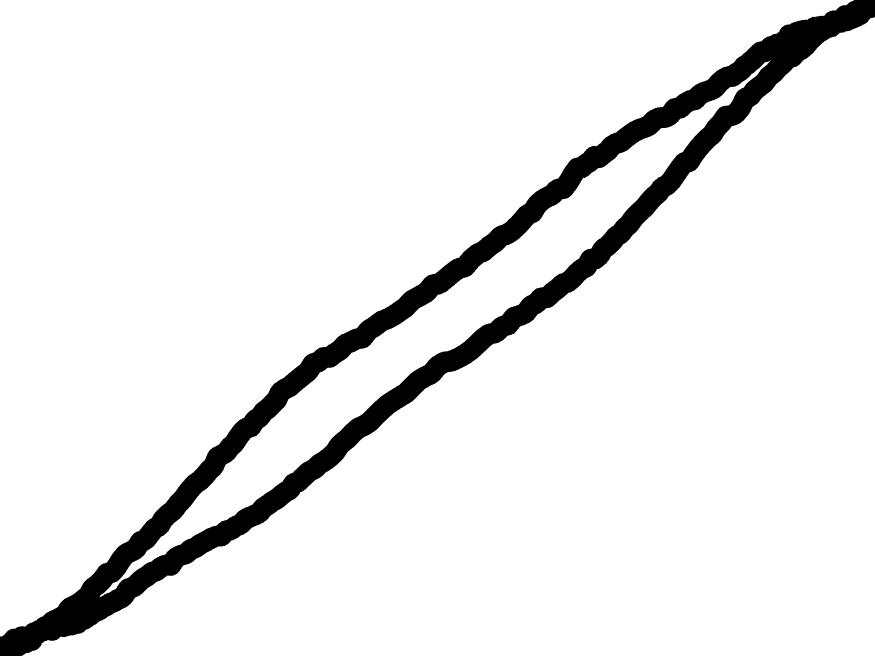

Supplement: S1 Data — (ZIP) [file pone.0312954.s001.zip › S1 data/1.V-I trajectories/0/1 highcool_1057/109_0.jpg]

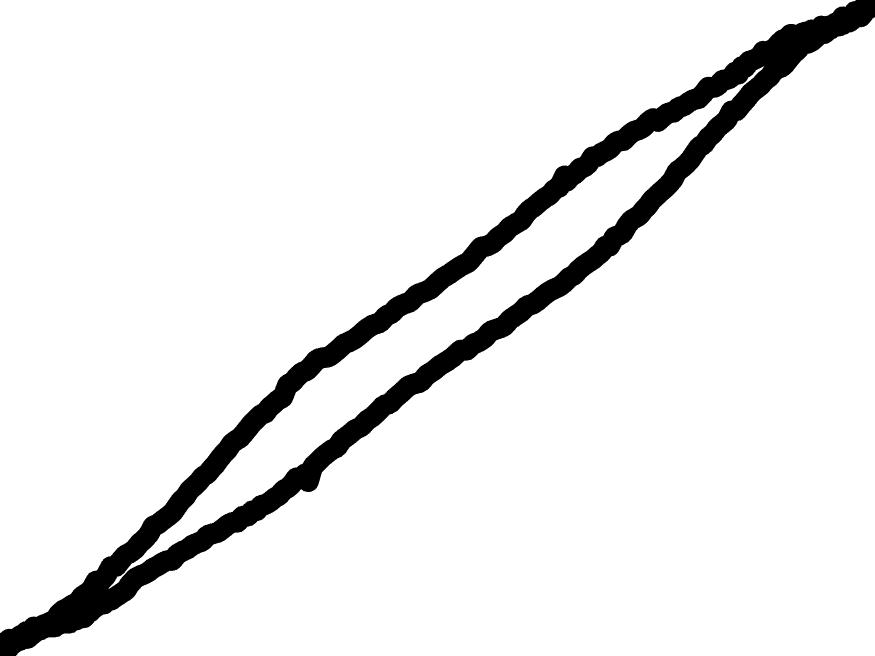

Supplement: S1 Data — (ZIP) [file pone.0312954.s001.zip › S1 data/1.V-I trajectories/0/1 highcool_1057/10_0.jpg]

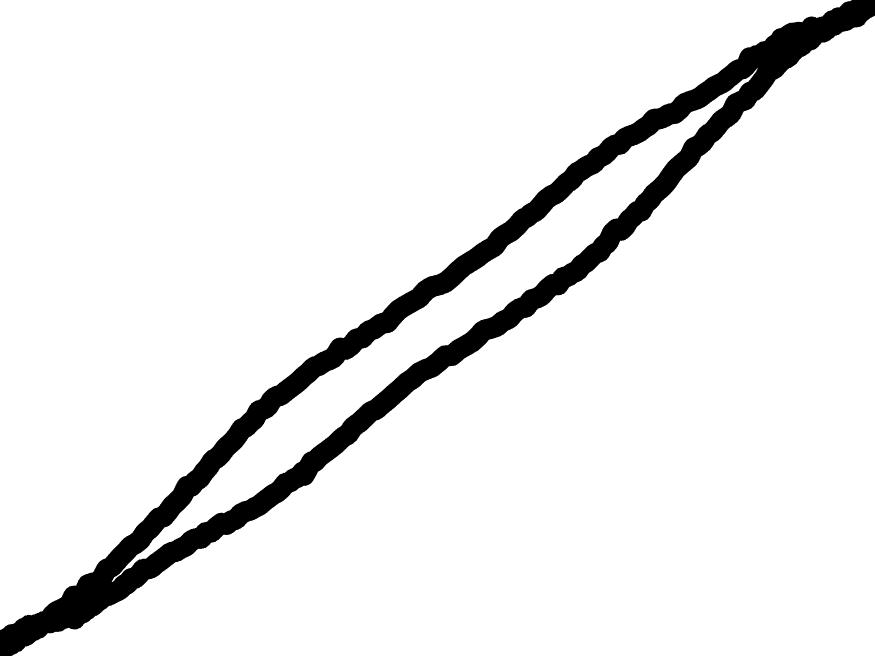

Supplement: S1 Data — (ZIP) [file pone.0312954.s001.zip › S1 data/1.V-I trajectories/0/1 highcool_1057/113_0.jpg]

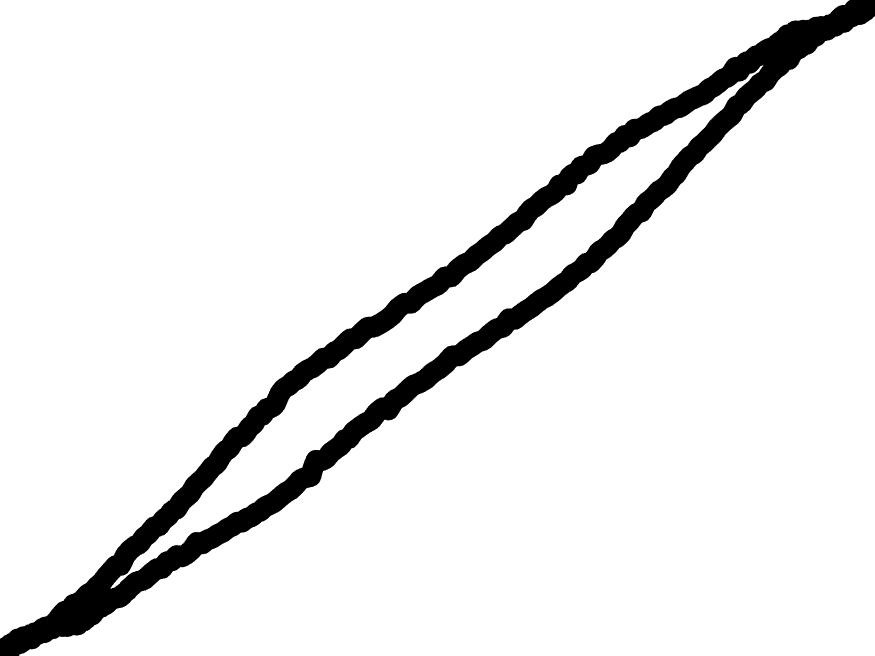

Supplement: S1 Data — (ZIP) [file pone.0312954.s001.zip › S1 data/1.V-I trajectories/0/1 highcool_1057/117_0.jpg]

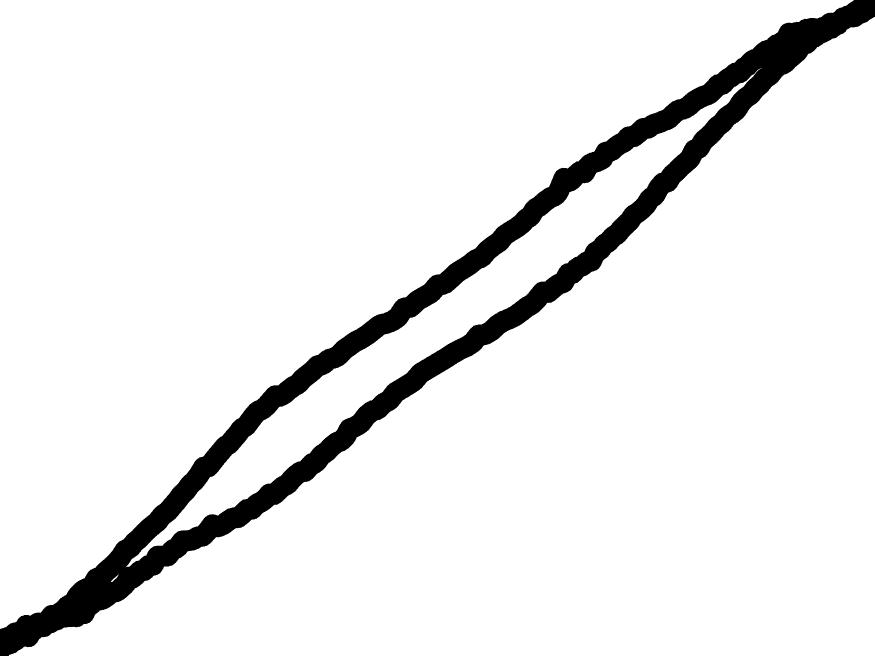

Supplement: S1 Data — (ZIP) [file pone.0312954.s001.zip › S1 data/1.V-I trajectories/0/1 highcool_1057/121_0.jpg]

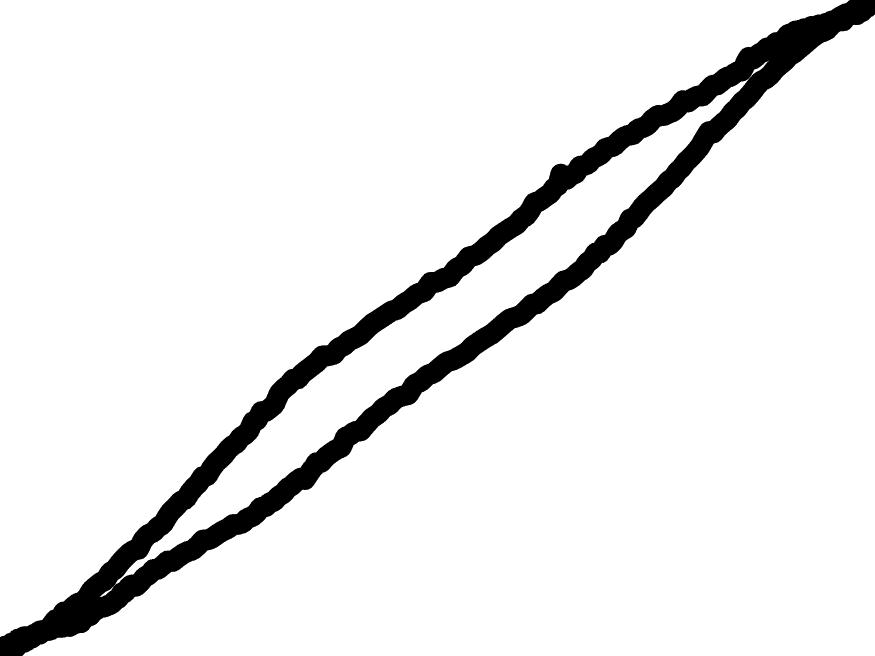

Supplement: S1 Data — (ZIP) [file pone.0312954.s001.zip › S1 data/1.V-I trajectories/0/1 highcool_1057/125_0.jpg]

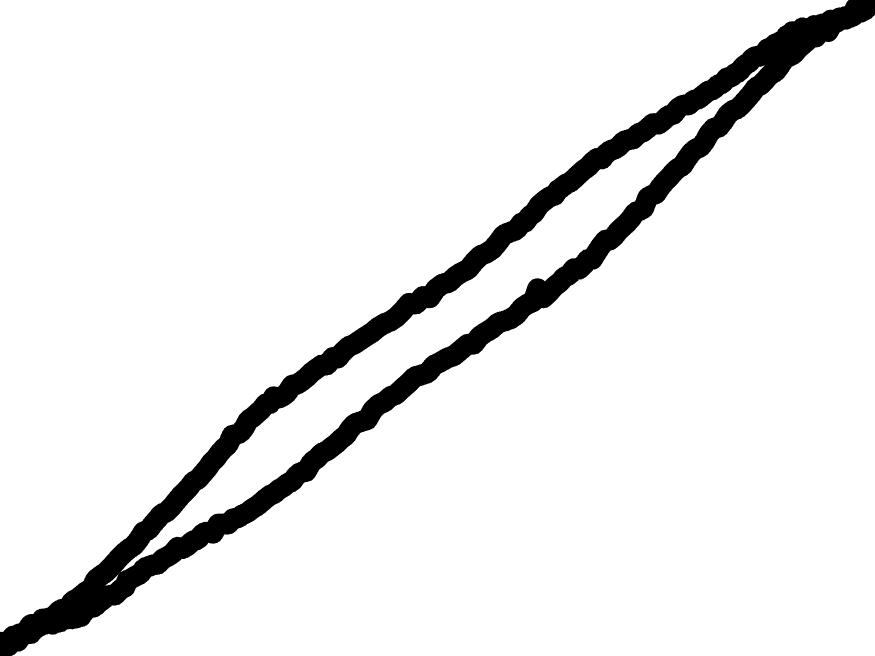

Supplement: S1 Data — (ZIP) [file pone.0312954.s001.zip › S1 data/1.V-I trajectories/0/1 highcool_1057/129_0.jpg]

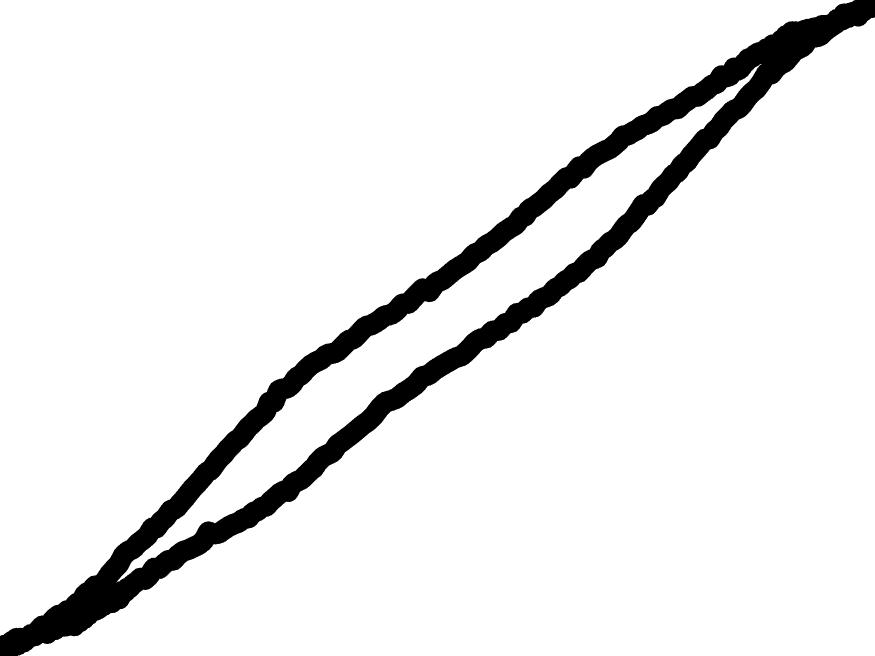

Supplement: S1 Data — (ZIP) [file pone.0312954.s001.zip › S1 data/1.V-I trajectories/0/1 highcool_1057/133_0.jpg]

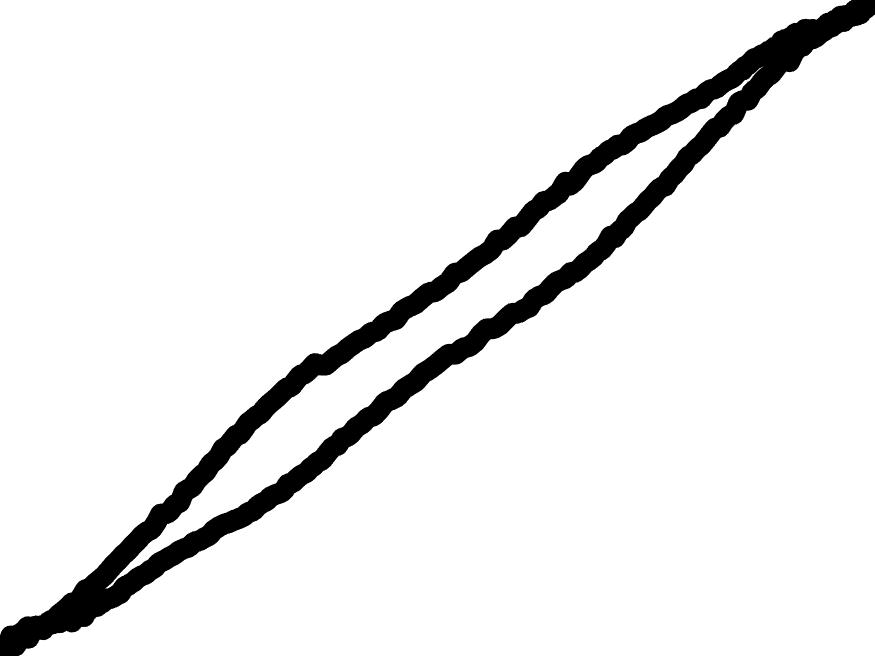

Supplement: S1 Data — (ZIP) [file pone.0312954.s001.zip › S1 data/1.V-I trajectories/0/1 highcool_1057/137_0.jpg]

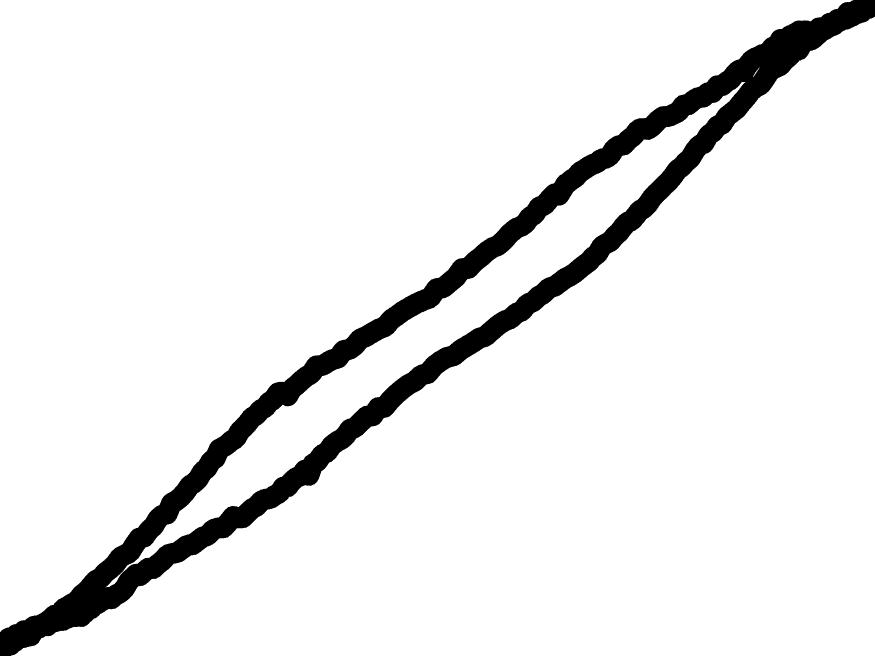

Supplement: S1 Data — (ZIP) [file pone.0312954.s001.zip › S1 data/1.V-I trajectories/0/1 highcool_1057/13_0.jpg]

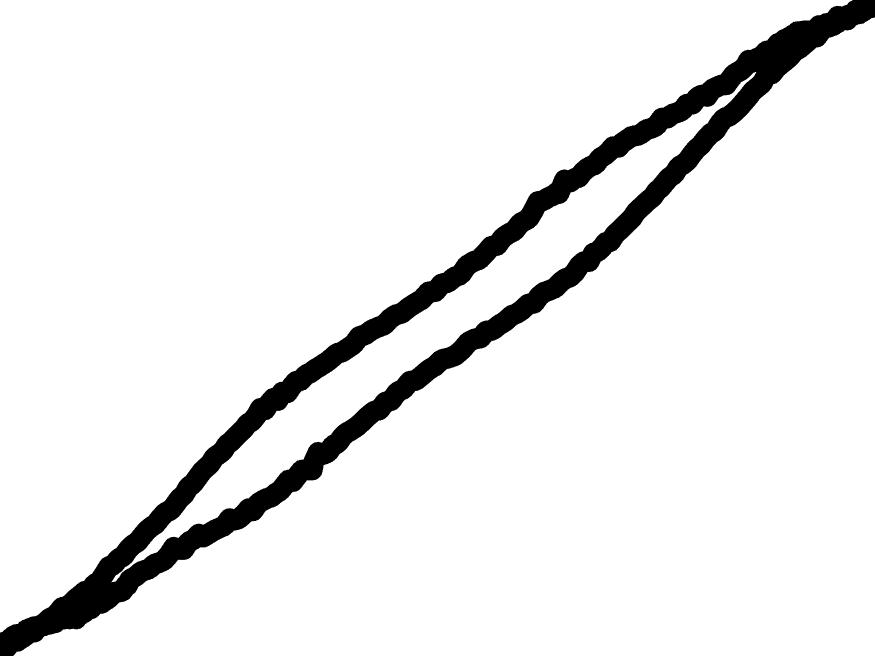

Supplement: S1 Data — (ZIP) [file pone.0312954.s001.zip › S1 data/1.V-I trajectories/0/1 highcool_1057/141_0.jpg]

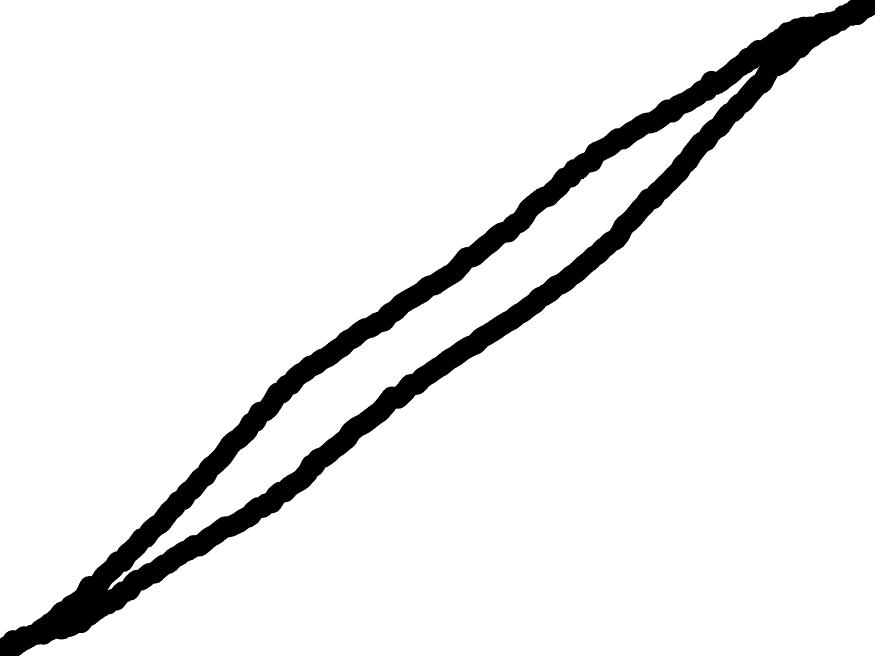

Supplement: S1 Data — (ZIP) [file pone.0312954.s001.zip › S1 data/1.V-I trajectories/0/1 highcool_1057/145_0.jpg]

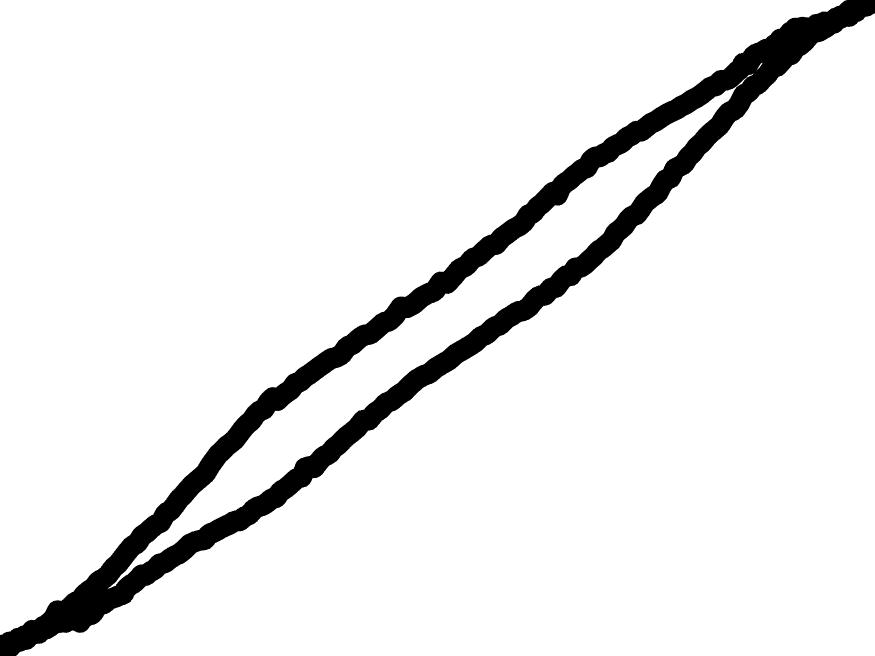

Supplement: S1 Data — (ZIP) [file pone.0312954.s001.zip › S1 data/1.V-I trajectories/0/1 highcool_1057/149_0.jpg]

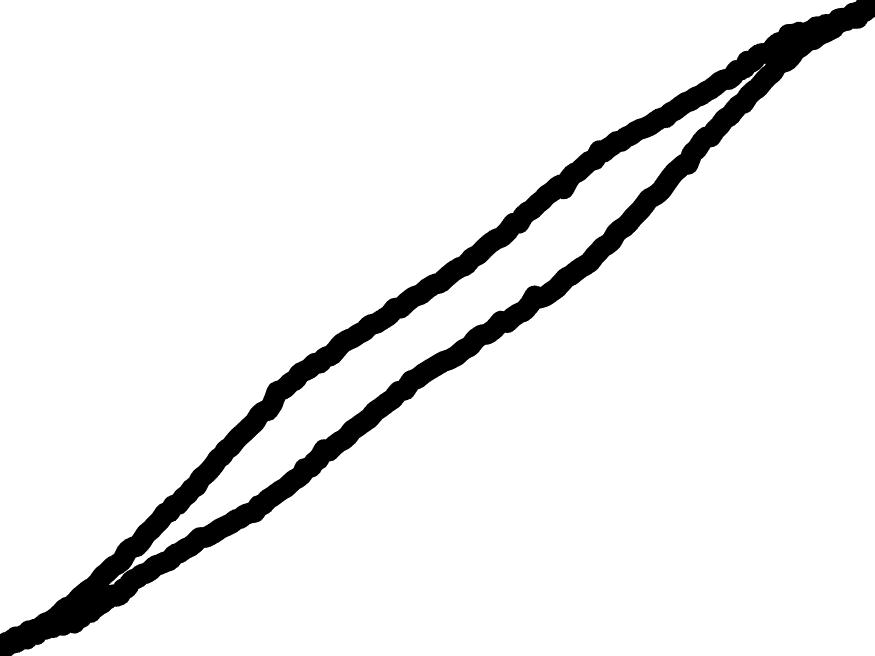

Supplement: S1 Data — (ZIP) [file pone.0312954.s001.zip › S1 data/1.V-I trajectories/0/1 highcool_1057/153_0.jpg]

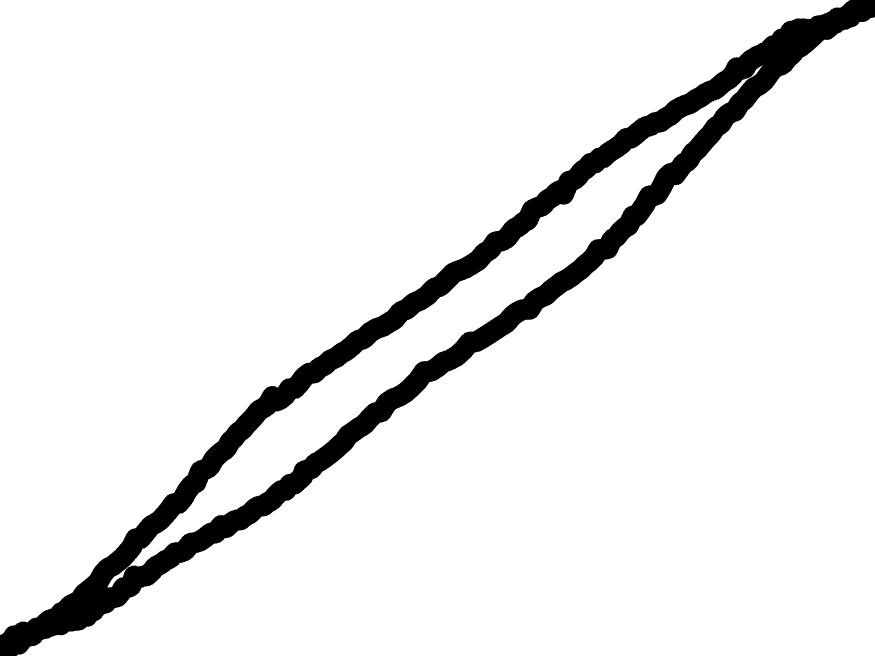

Supplement: S1 Data — (ZIP) [file pone.0312954.s001.zip › S1 data/1.V-I trajectories/0/1 highcool_1057/157_0.jpg]

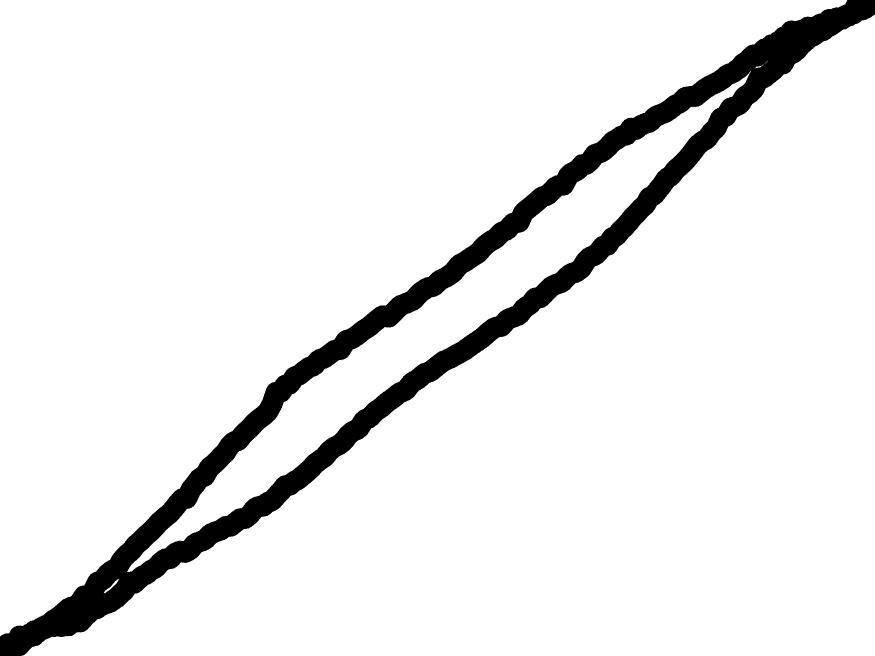

Supplement: S1 Data — (ZIP) [file pone.0312954.s001.zip › S1 data/1.V-I trajectories/0/1 highcool_1057/161_0.jpg]

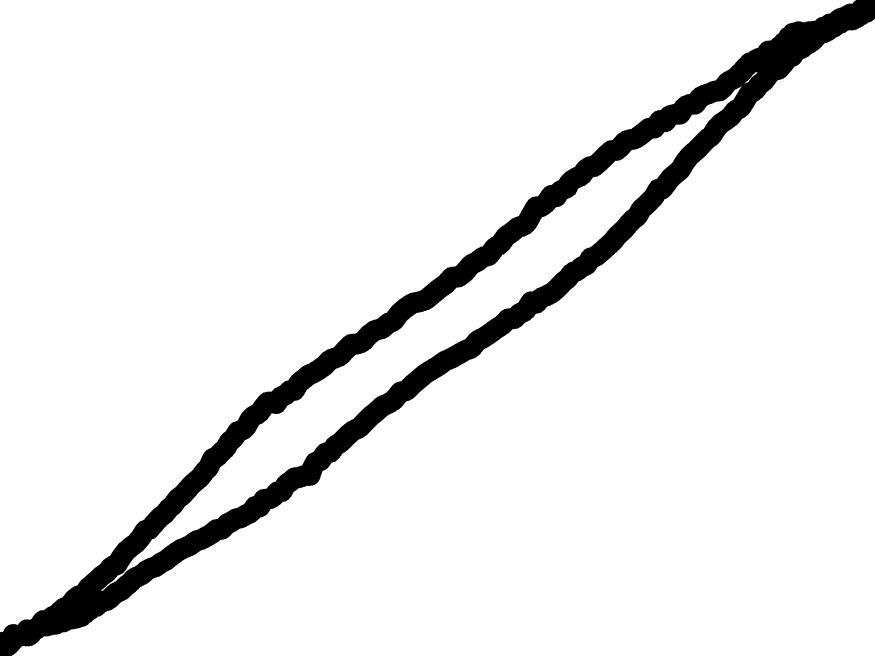

Supplement: S1 Data — (ZIP) [file pone.0312954.s001.zip › S1 data/1.V-I trajectories/0/1 highcool_1057/165_0.jpg]

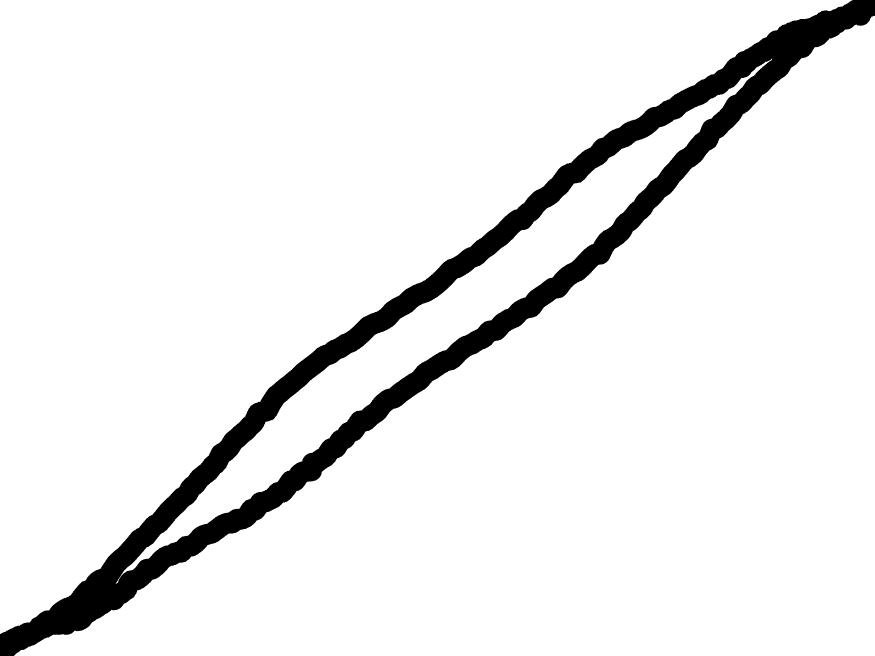

Supplement: S1 Data — (ZIP) [file pone.0312954.s001.zip › S1 data/1.V-I trajectories/0/1 highcool_1057/169_0.jpg]

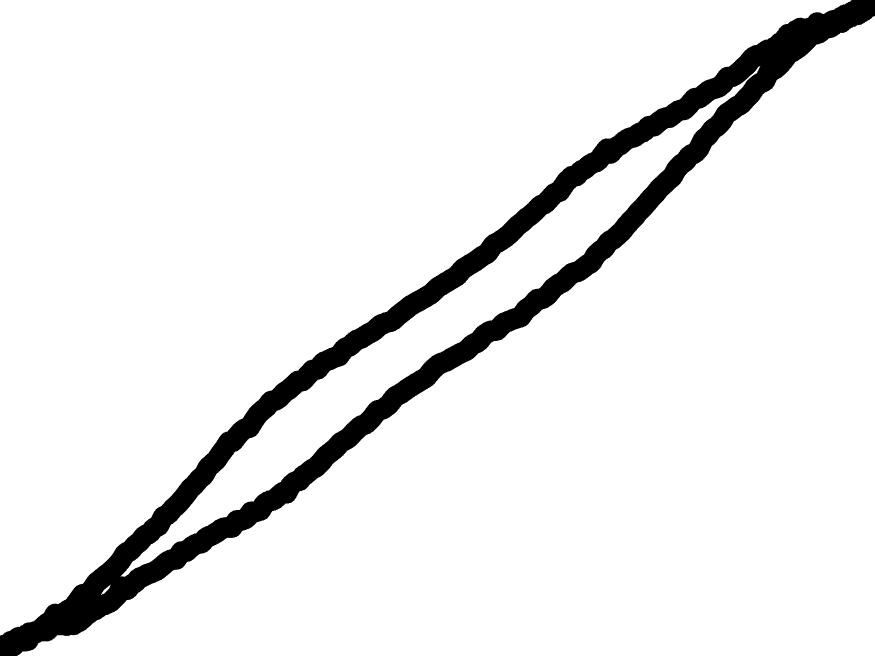

Supplement: S1 Data — (ZIP) [file pone.0312954.s001.zip › S1 data/1.V-I trajectories/0/1 highcool_1057/173_0.jpg]

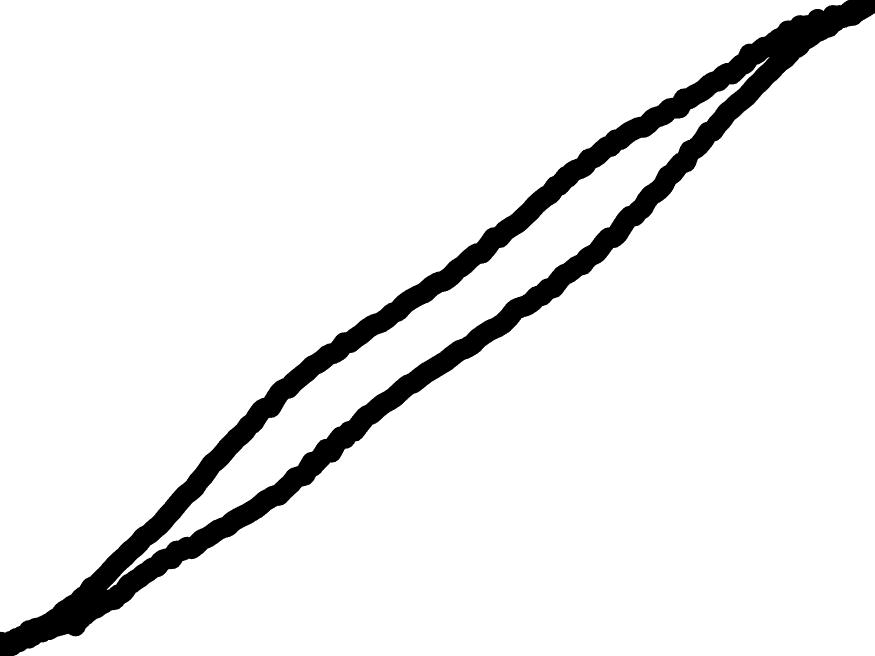

Supplement: S1 Data — (ZIP) [file pone.0312954.s001.zip › S1 data/1.V-I trajectories/0/1 highcool_1057/177_0.jpg]

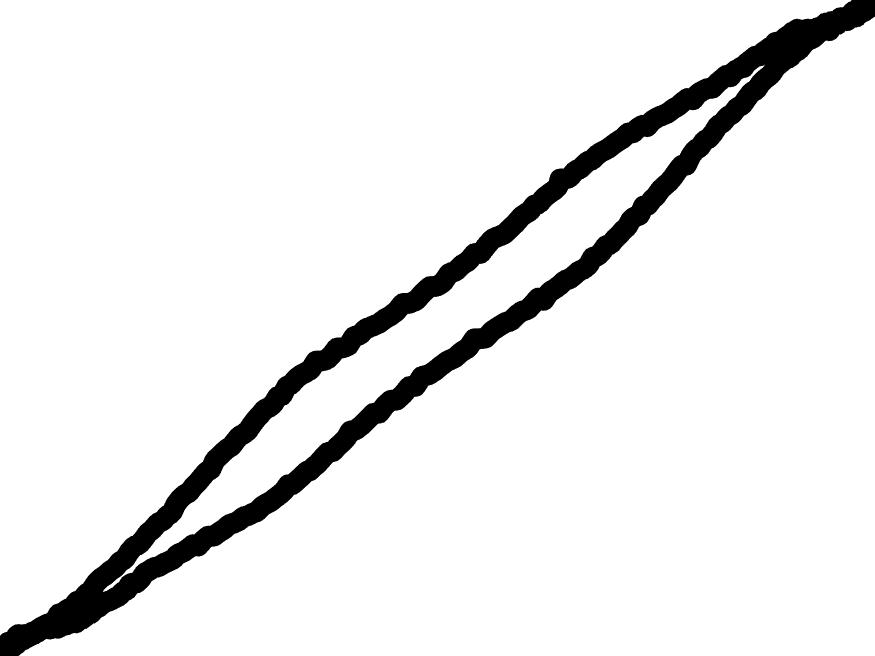

Supplement: S1 Data — (ZIP) [file pone.0312954.s001.zip › S1 data/1.V-I trajectories/0/1 highcool_1057/17_0.jpg]

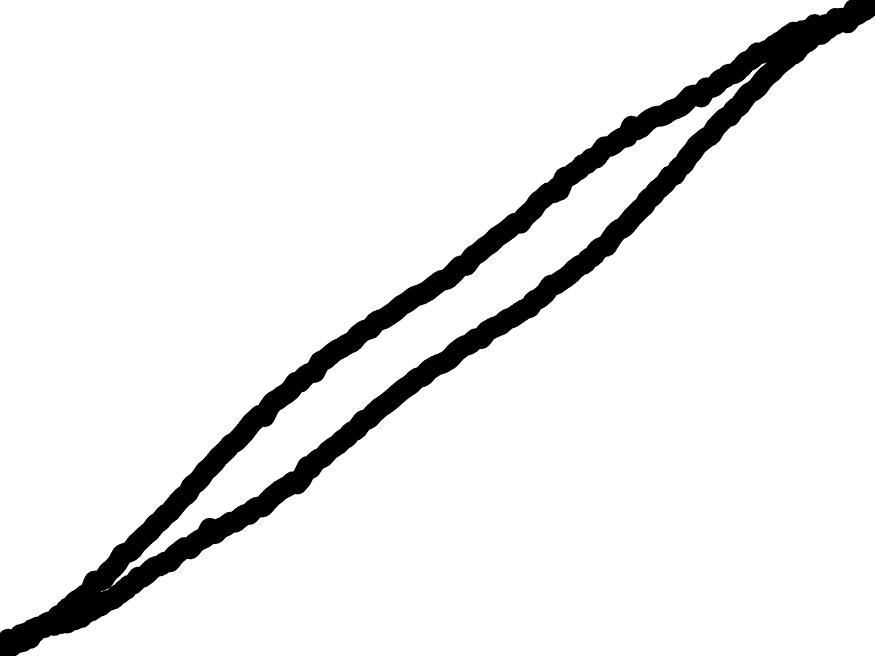

Supplement: S1 Data — (ZIP) [file pone.0312954.s001.zip › S1 data/1.V-I trajectories/0/1 highcool_1057/181_0.jpg]

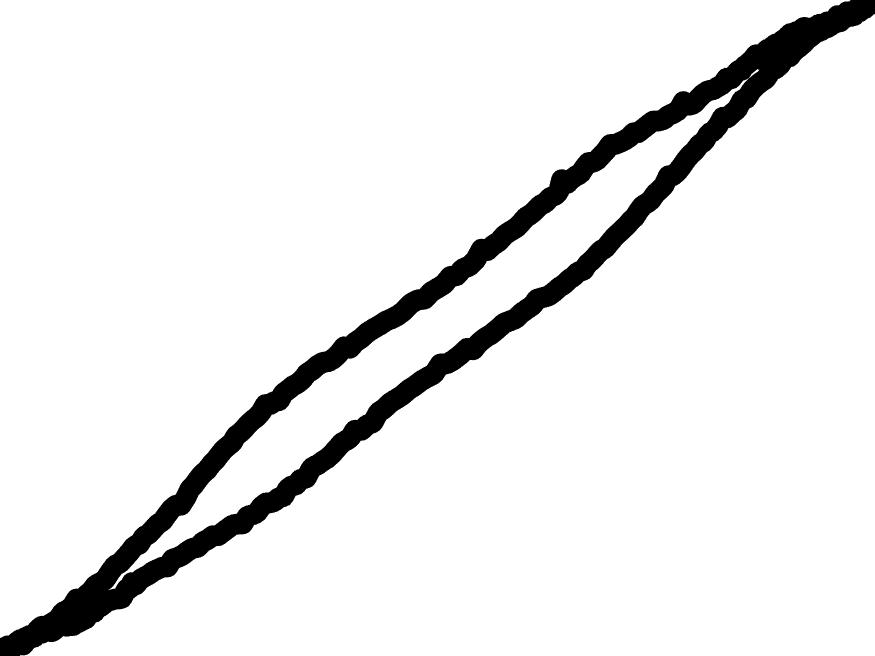

Supplement: S1 Data — (ZIP) [file pone.0312954.s001.zip › S1 data/1.V-I trajectories/0/1 highcool_1057/185_0.jpg]

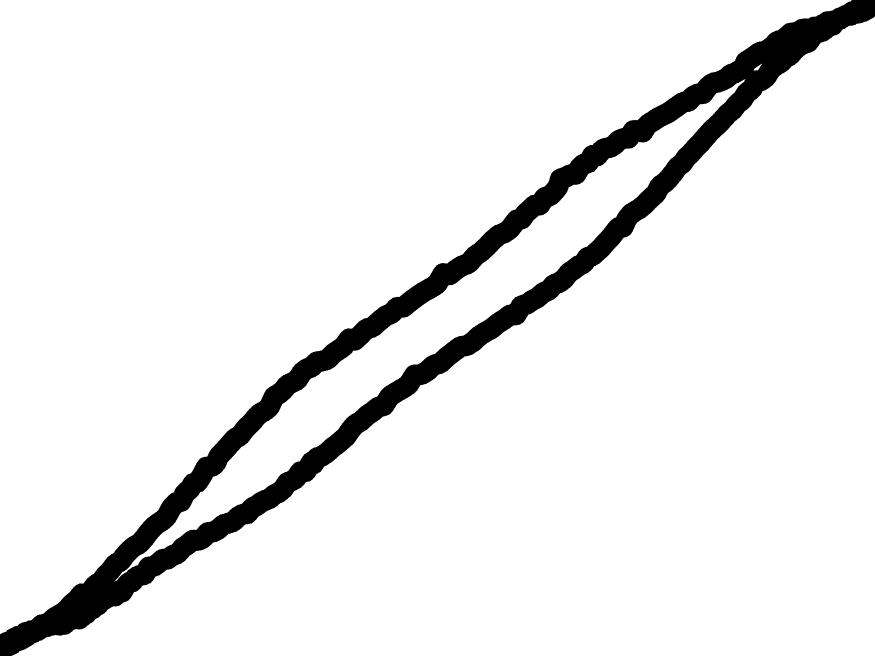

Supplement: S1 Data — (ZIP) [file pone.0312954.s001.zip › S1 data/1.V-I trajectories/0/1 highcool_1057/189_0.jpg]

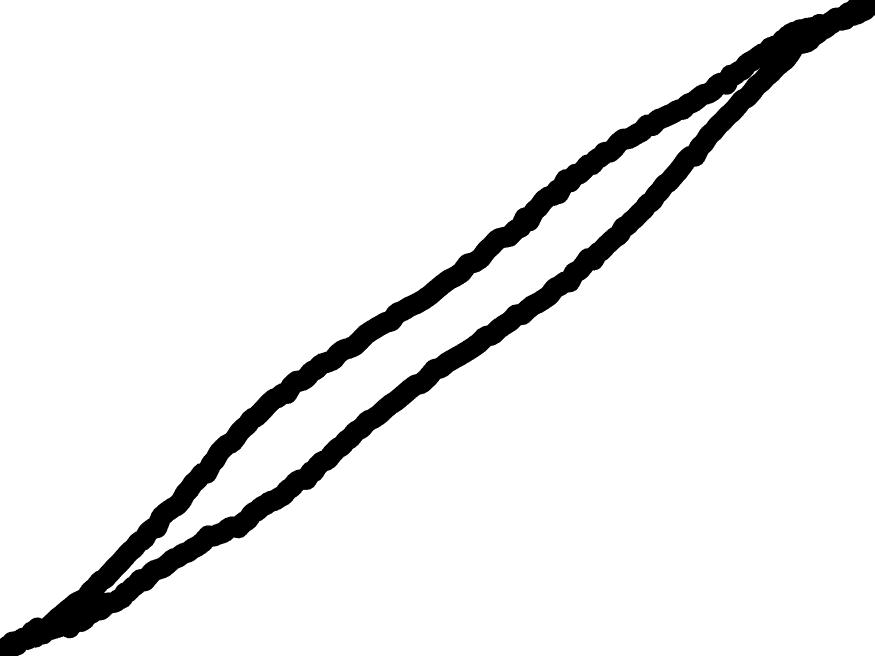

Supplement: S1 Data — (ZIP) [file pone.0312954.s001.zip › S1 data/1.V-I trajectories/0/1 highcool_1057/193_0.jpg]

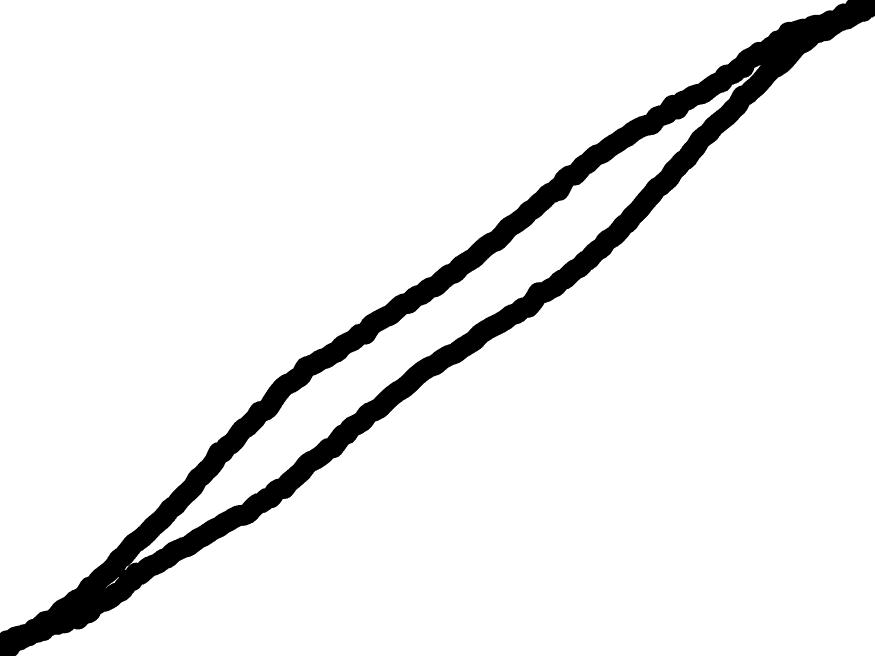

Supplement: S1 Data — (ZIP) [file pone.0312954.s001.zip › S1 data/1.V-I trajectories/0/1 highcool_1057/197_0.jpg]

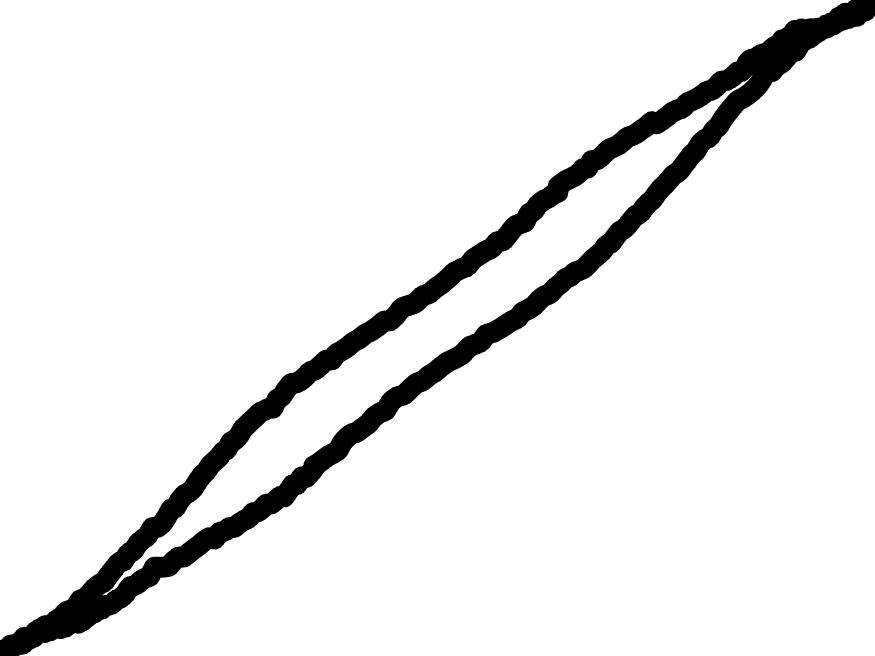

Supplement: S1 Data — (ZIP) [file pone.0312954.s001.zip › S1 data/1.V-I trajectories/0/1 highcool_1057/201_0.jpg]

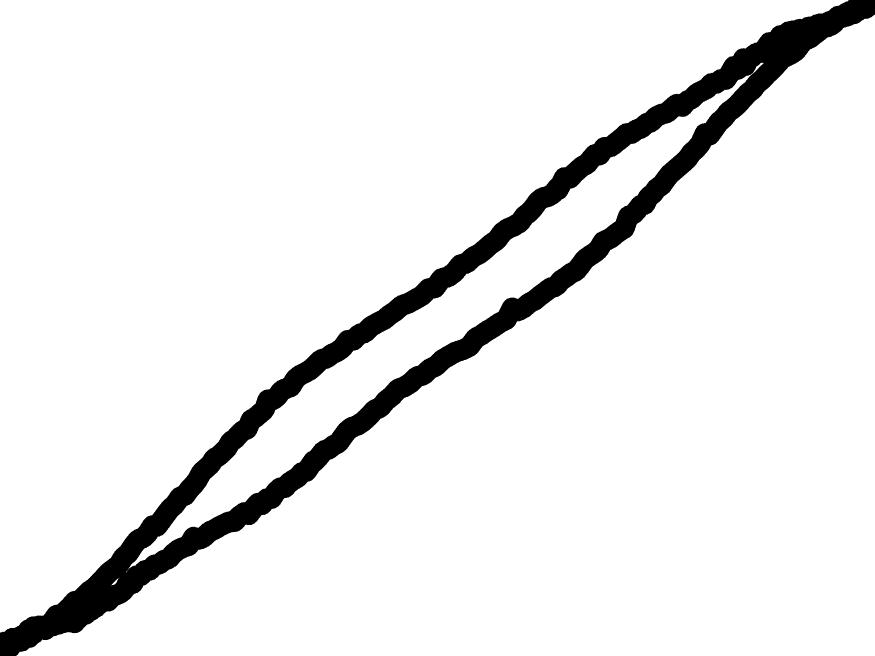

Supplement: S1 Data — (ZIP) [file pone.0312954.s001.zip › S1 data/1.V-I trajectories/0/1 highcool_1057/205_0.jpg]

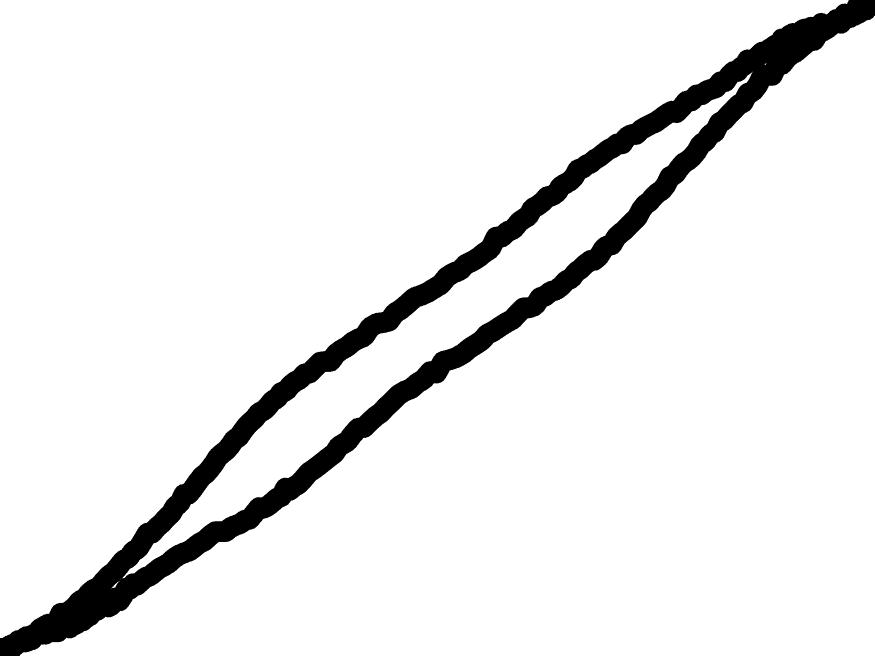

Supplement: S1 Data — (ZIP) [file pone.0312954.s001.zip › S1 data/1.V-I trajectories/0/1 highcool_1057/209_0.jpg]

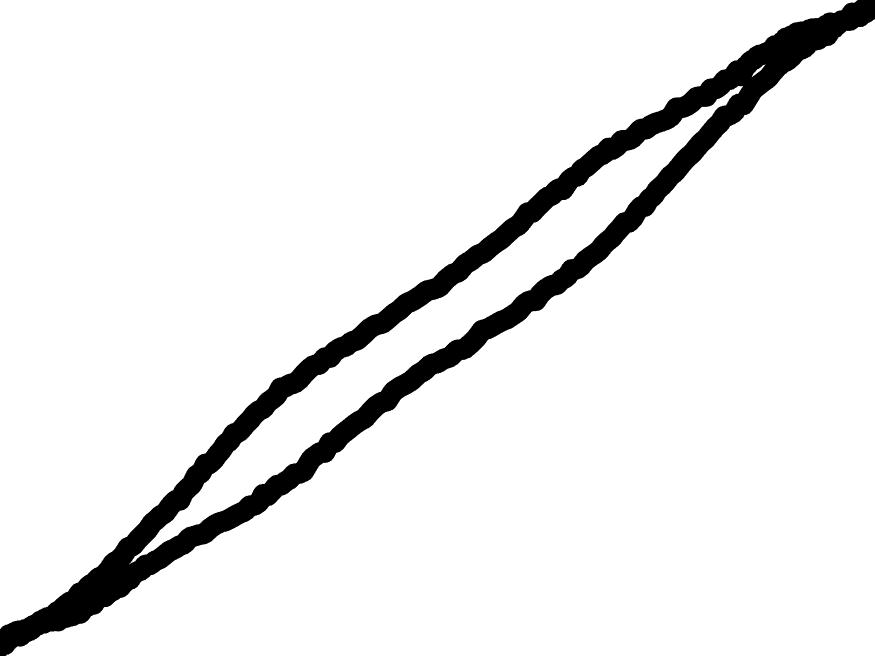

Supplement: S1 Data — (ZIP) [file pone.0312954.s001.zip › S1 data/1.V-I trajectories/0/1 highcool_1057/213_0.jpg]

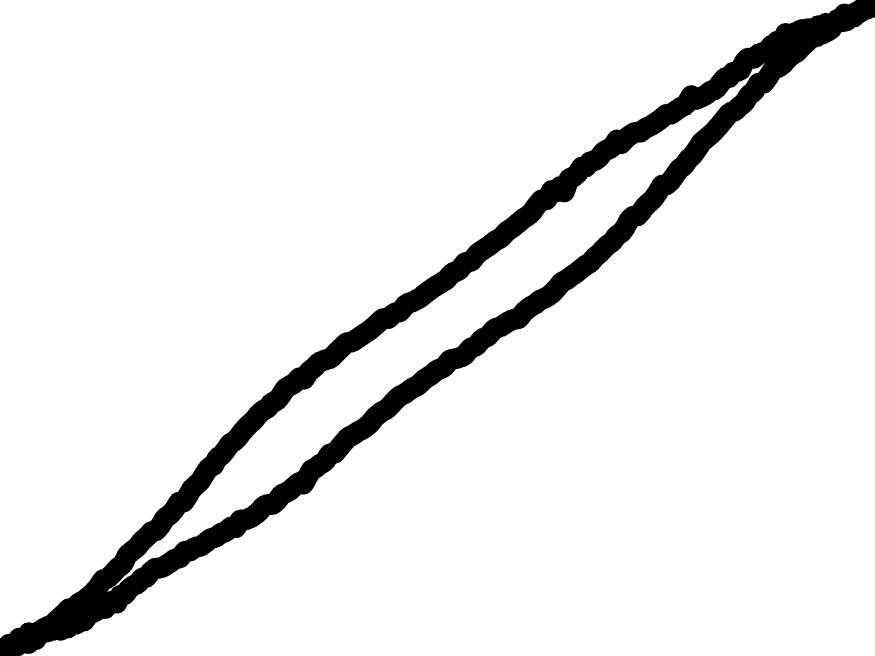

Supplement: S1 Data — (ZIP) [file pone.0312954.s001.zip › S1 data/1.V-I trajectories/0/1 highcool_1057/217_0.jpg]

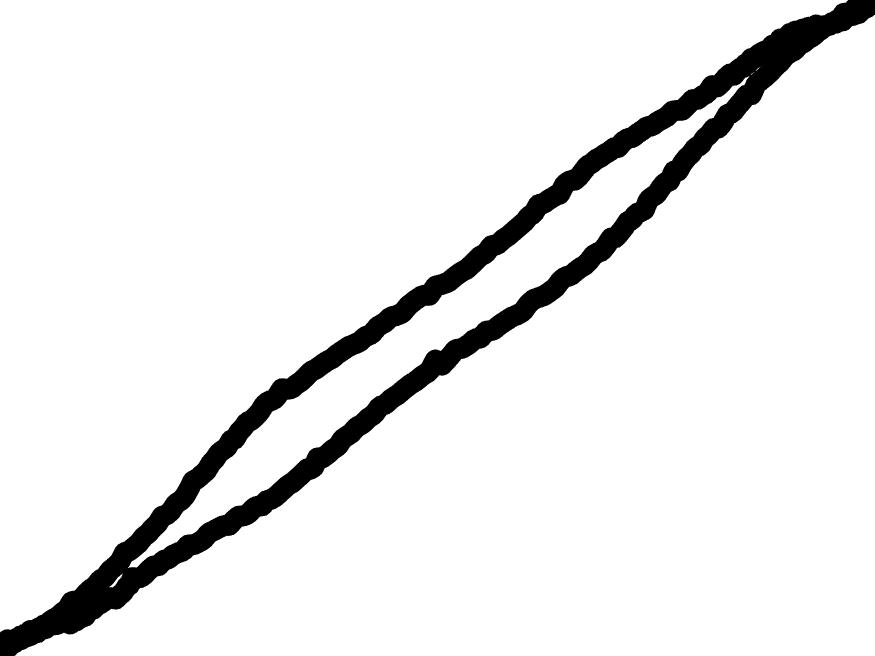

Supplement: S1 Data — (ZIP) [file pone.0312954.s001.zip › S1 data/1.V-I trajectories/0/1 highcool_1057/21_0.jpg]

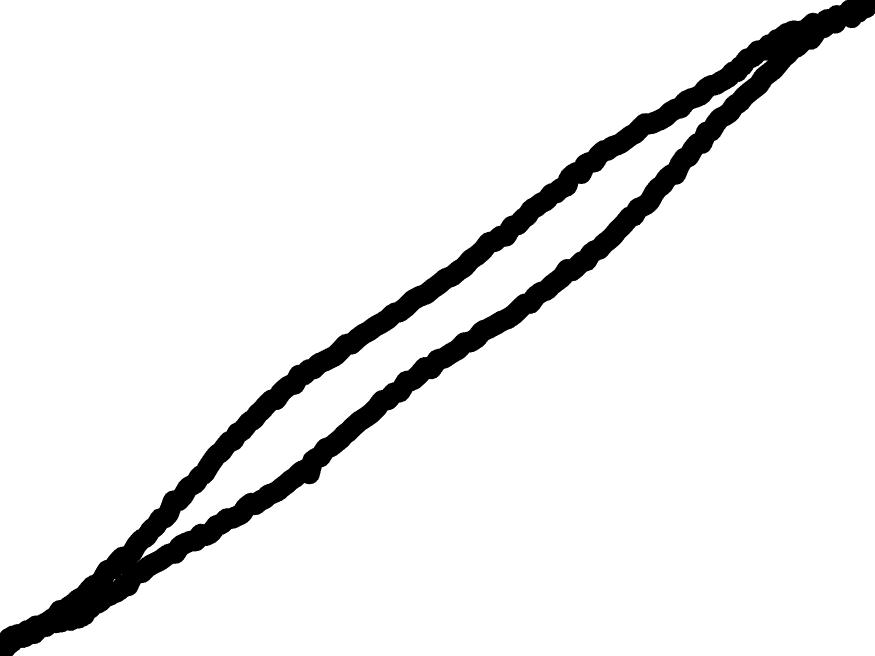

Supplement: S1 Data — (ZIP) [file pone.0312954.s001.zip › S1 data/1.V-I trajectories/0/1 highcool_1057/221_0.jpg]

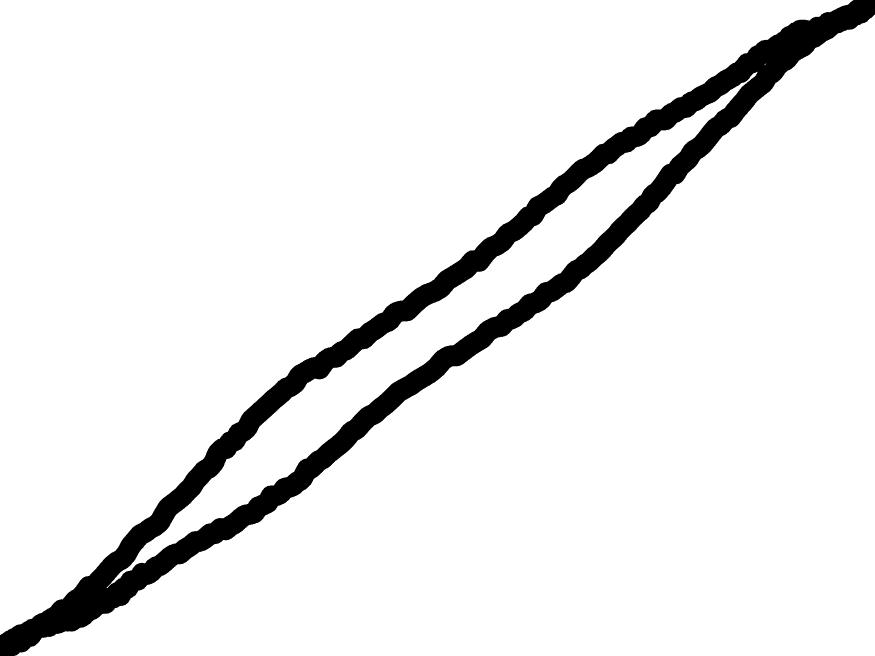

Supplement: S1 Data — (ZIP) [file pone.0312954.s001.zip › S1 data/1.V-I trajectories/0/1 highcool_1057/225_0.jpg]

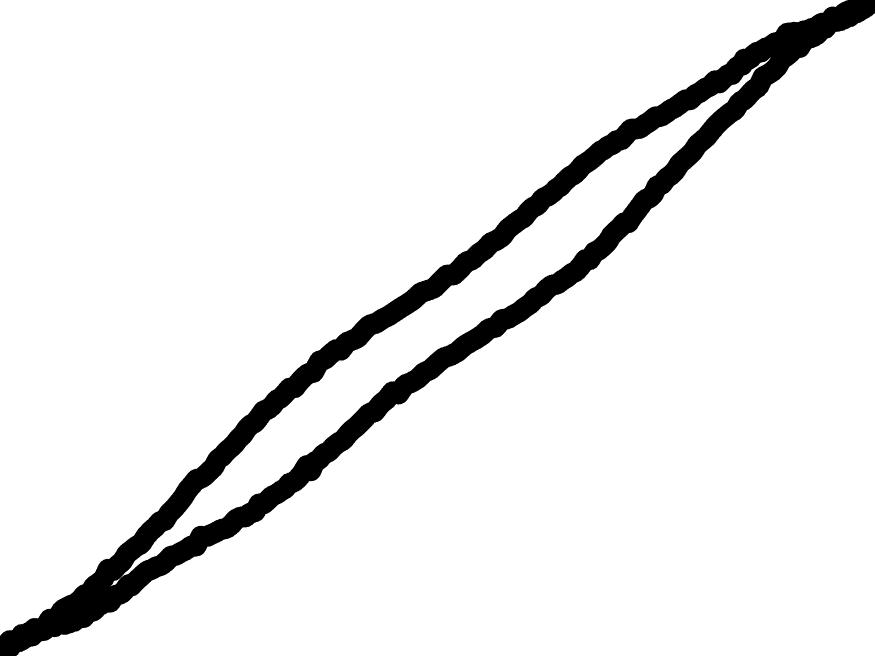

Supplement: S1 Data — (ZIP) [file pone.0312954.s001.zip › S1 data/1.V-I trajectories/0/1 highcool_1057/229_0.jpg]

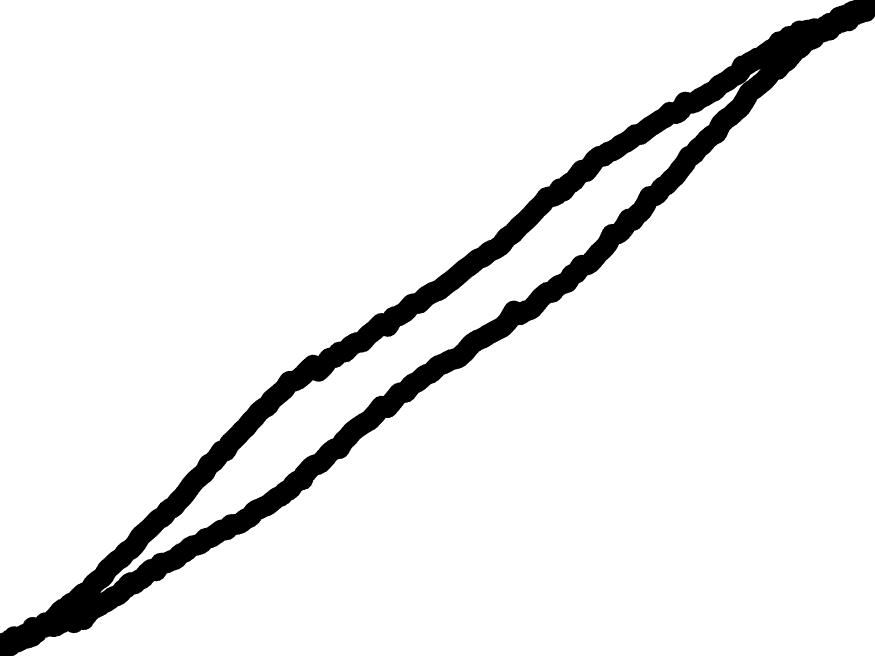

Supplement: S1 Data — (ZIP) [file pone.0312954.s001.zip › S1 data/1.V-I trajectories/0/1 highcool_1057/233_0.jpg]

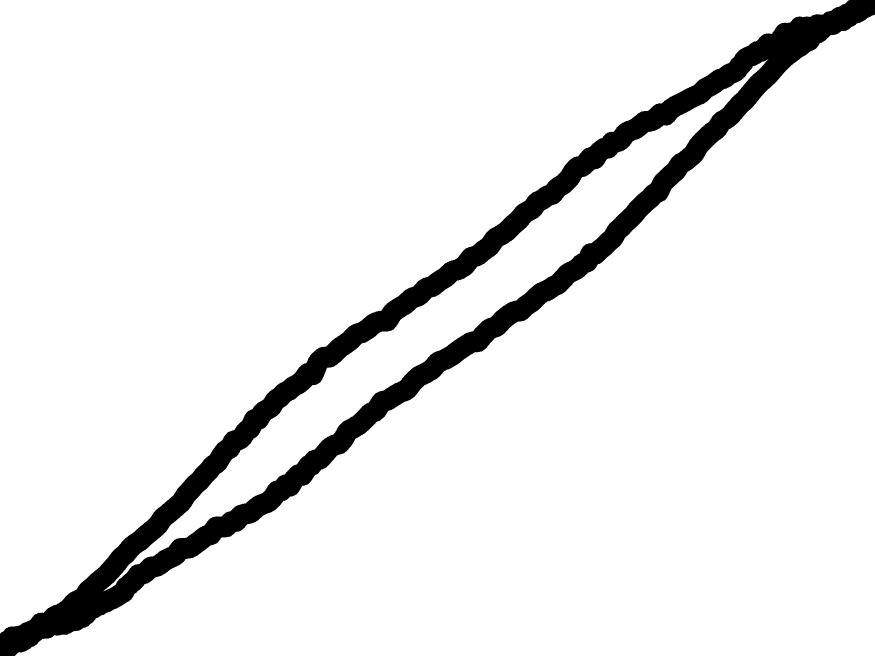

Supplement: S1 Data — (ZIP) [file pone.0312954.s001.zip › S1 data/1.V-I trajectories/0/1 highcool_1057/237_0.jpg]

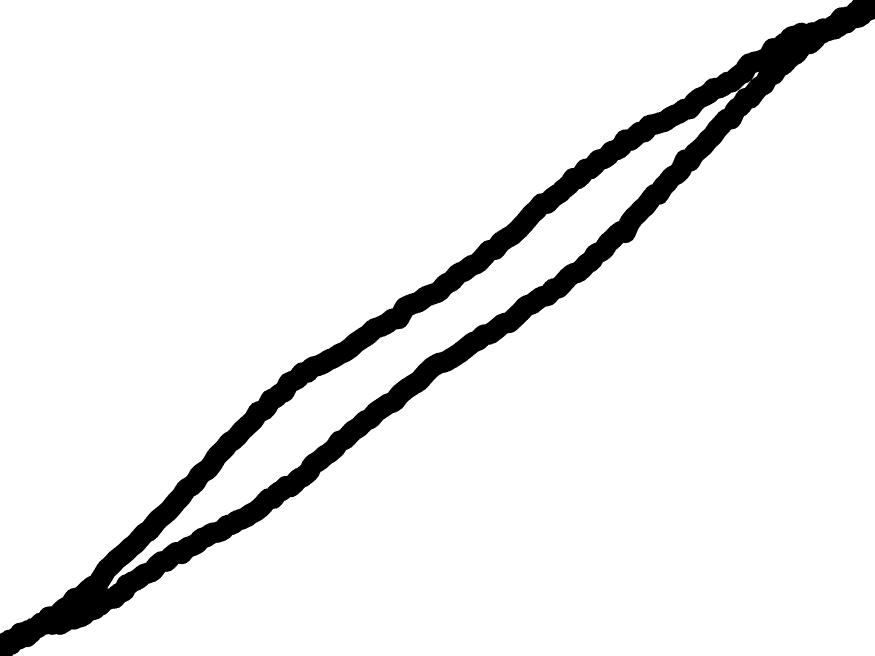

Supplement: S1 Data — (ZIP) [file pone.0312954.s001.zip › S1 data/1.V-I trajectories/0/1 highcool_1057/241_0.jpg]

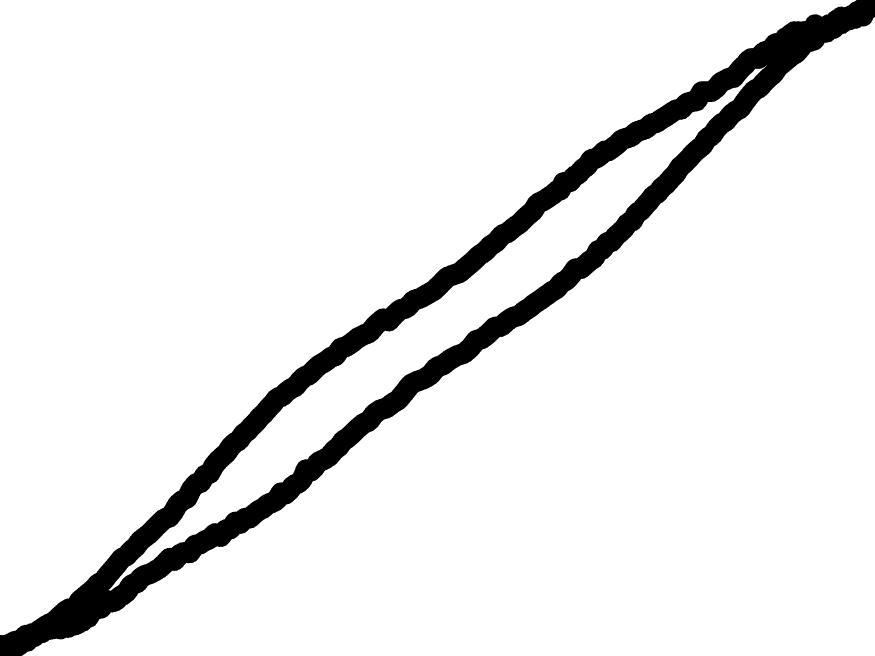

Supplement: S1 Data — (ZIP) [file pone.0312954.s001.zip › S1 data/1.V-I trajectories/0/1 highcool_1057/245_0.jpg]

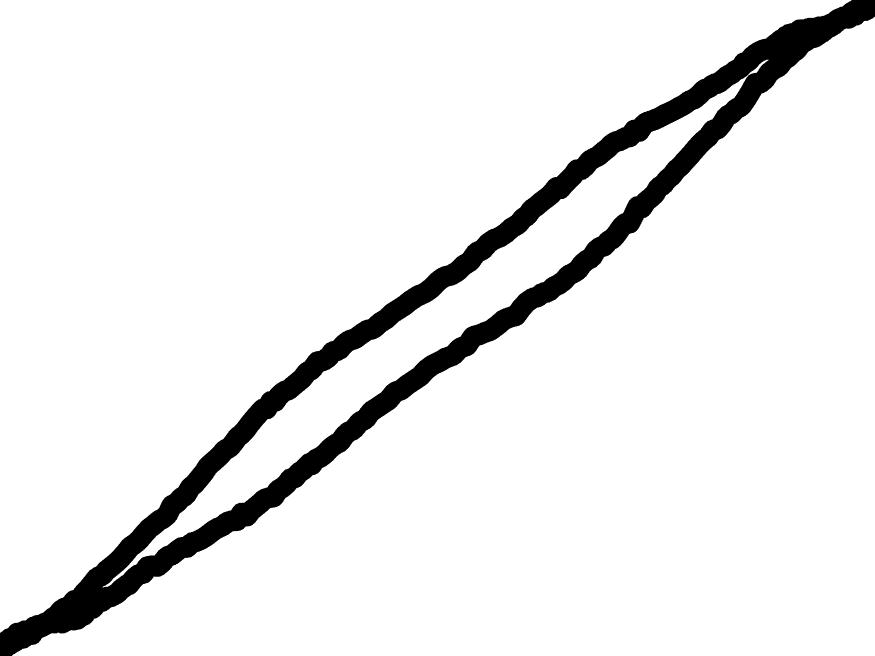

Supplement: S1 Data — (ZIP) [file pone.0312954.s001.zip › S1 data/1.V-I trajectories/0/1 highcool_1057/249_0.jpg]

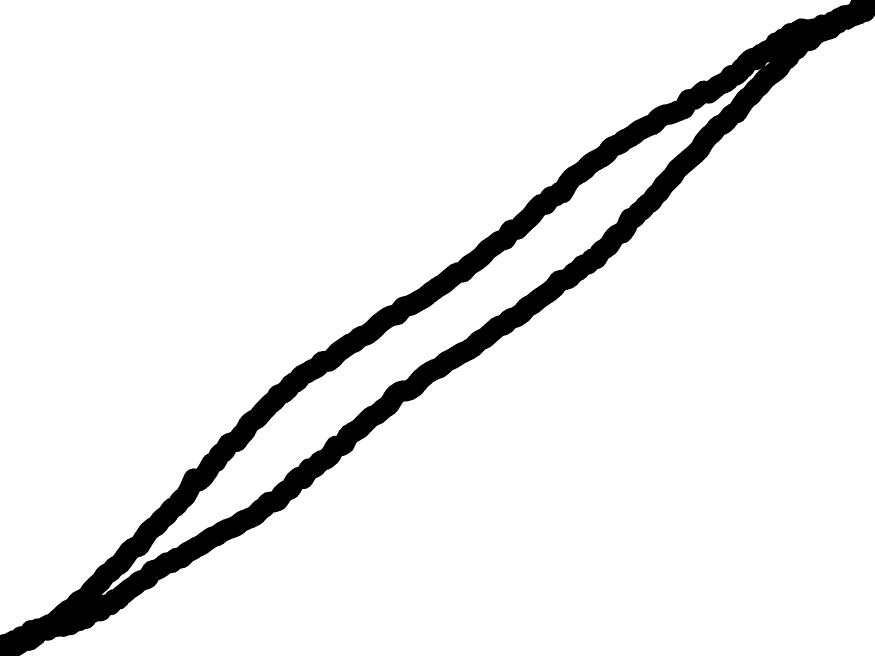

Supplement: S1 Data — (ZIP) [file pone.0312954.s001.zip › S1 data/1.V-I trajectories/0/1 highcool_1057/253_0.jpg]

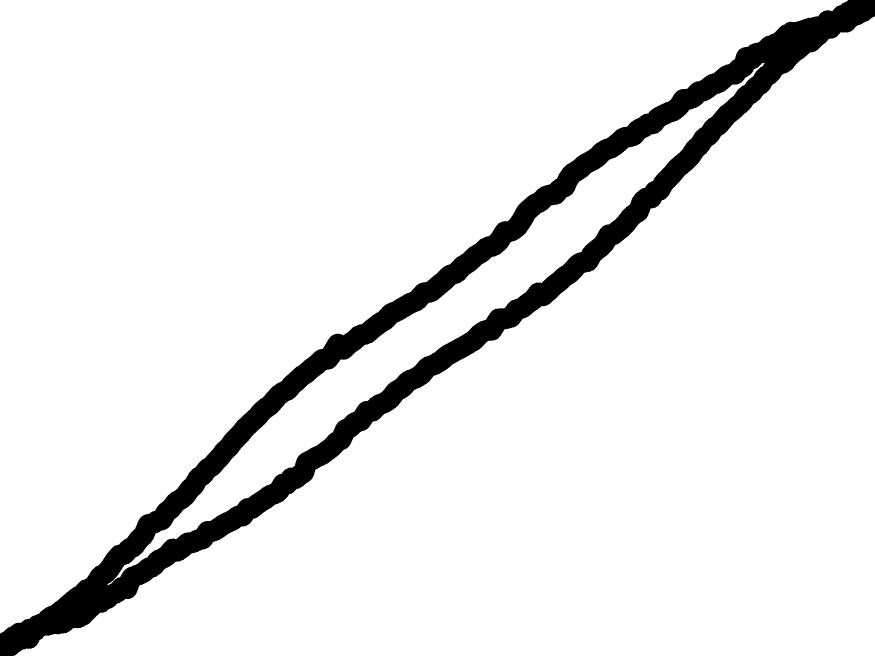

Supplement: S1 Data — (ZIP) [file pone.0312954.s001.zip › S1 data/1.V-I trajectories/0/1 highcool_1057/257_0.jpg]

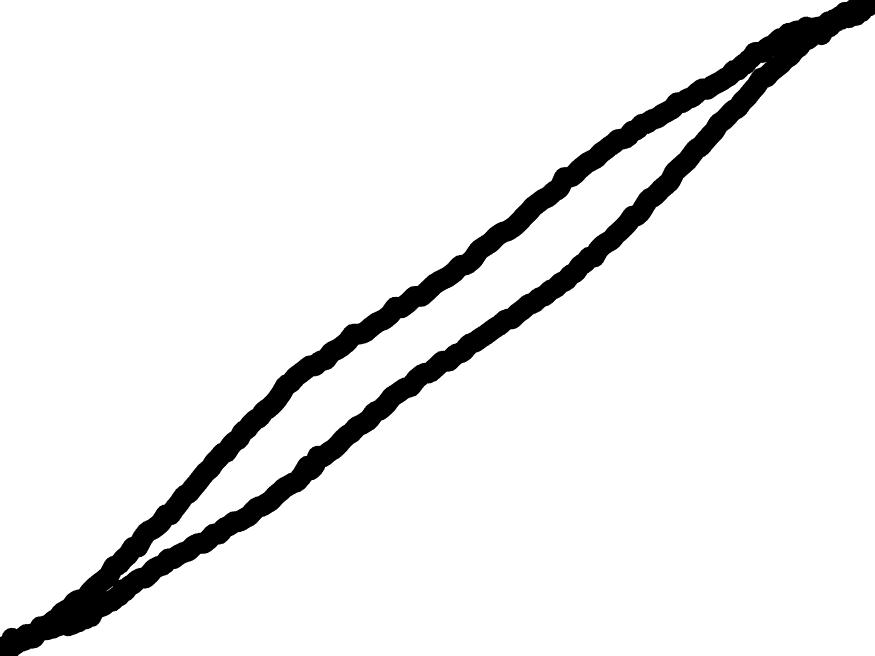

Supplement: S1 Data — (ZIP) [file pone.0312954.s001.zip › S1 data/1.V-I trajectories/0/1 highcool_1057/25_0.jpg]

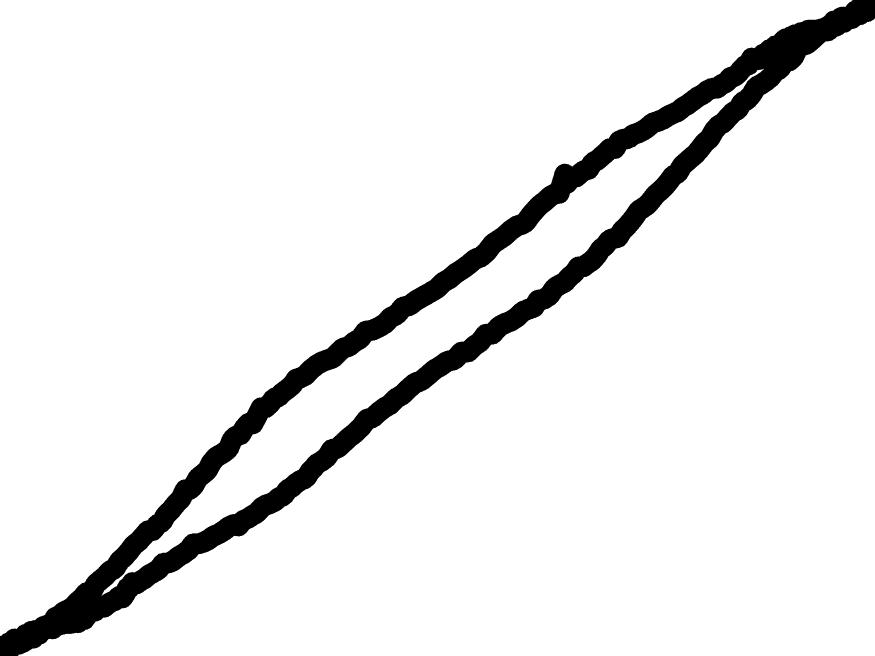

Supplement: S1 Data — (ZIP) [file pone.0312954.s001.zip › S1 data/1.V-I trajectories/0/1 highcool_1057/261_0.jpg]

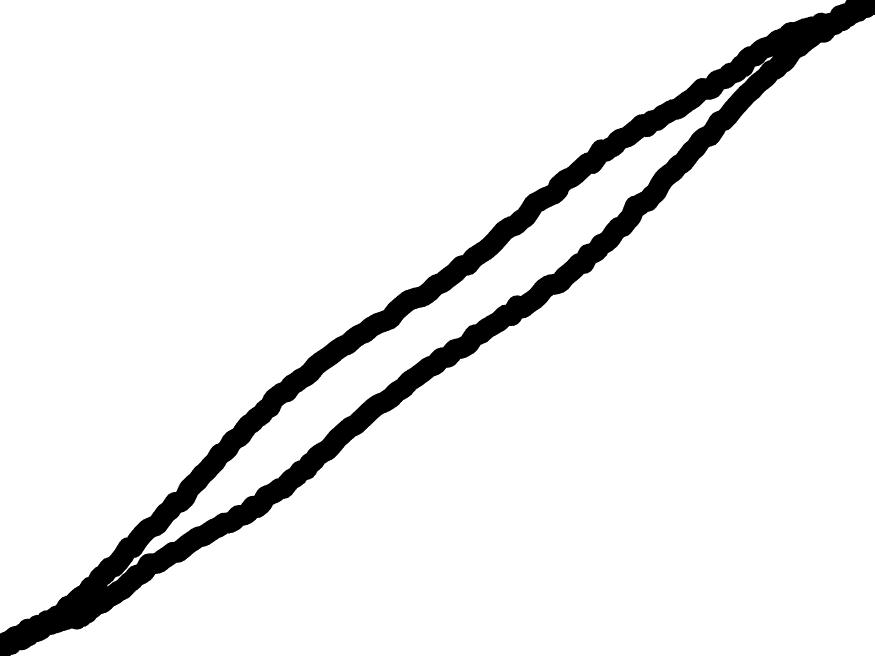

Supplement: S1 Data — (ZIP) [file pone.0312954.s001.zip › S1 data/1.V-I trajectories/0/1 highcool_1057/265_0.jpg]

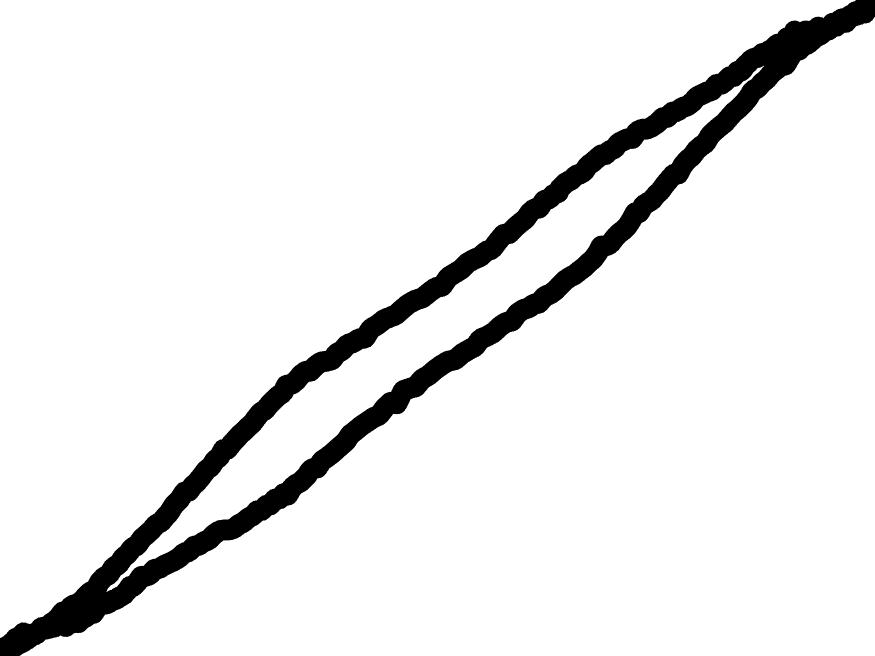

Supplement: S1 Data — (ZIP) [file pone.0312954.s001.zip › S1 data/1.V-I trajectories/0/1 highcool_1057/269_0.jpg]

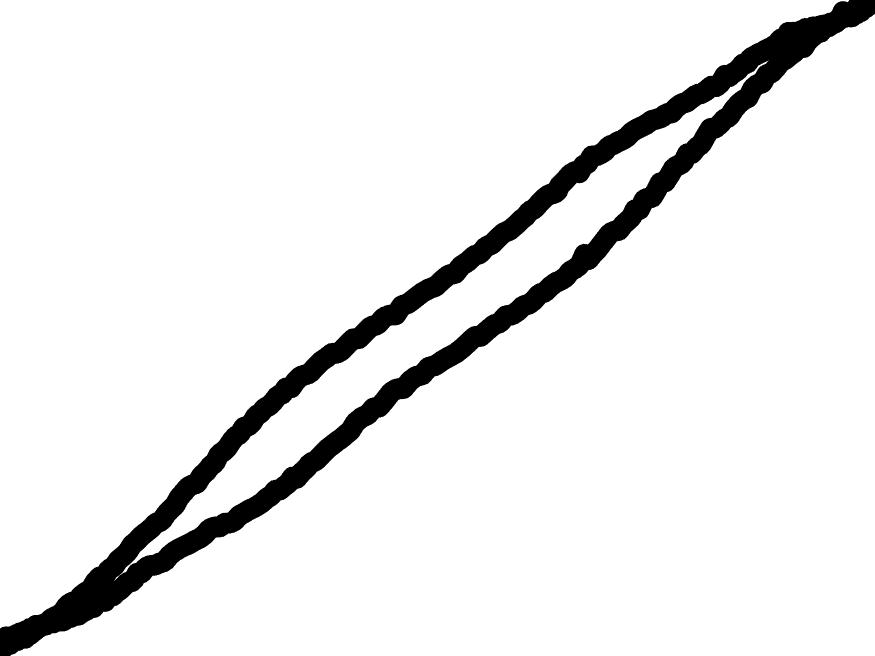

Supplement: S1 Data — (ZIP) [file pone.0312954.s001.zip › S1 data/1.V-I trajectories/0/1 highcool_1057/273_0.jpg]

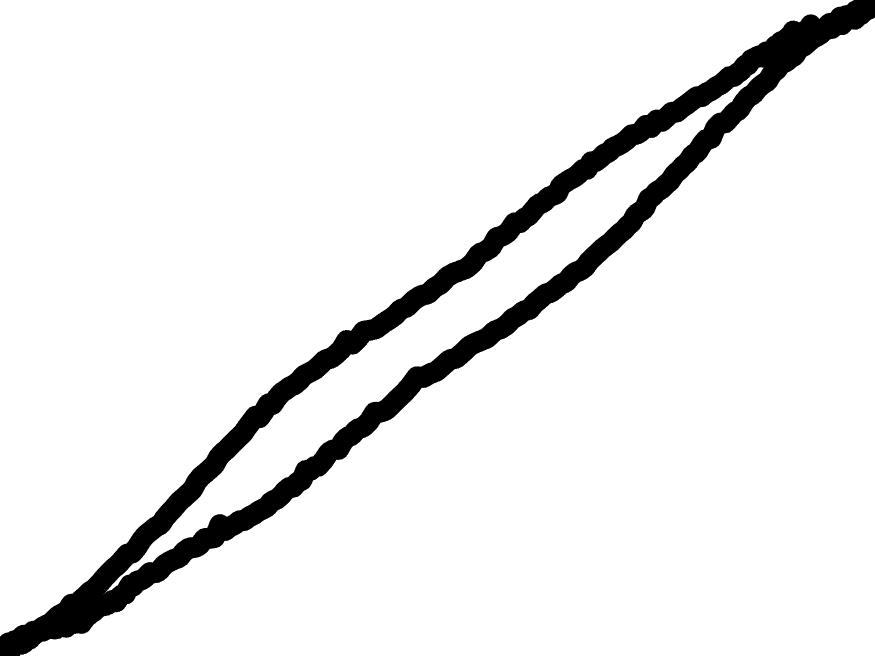

Supplement: S1 Data — (ZIP) [file pone.0312954.s001.zip › S1 data/1.V-I trajectories/0/1 highcool_1057/277_0.jpg]

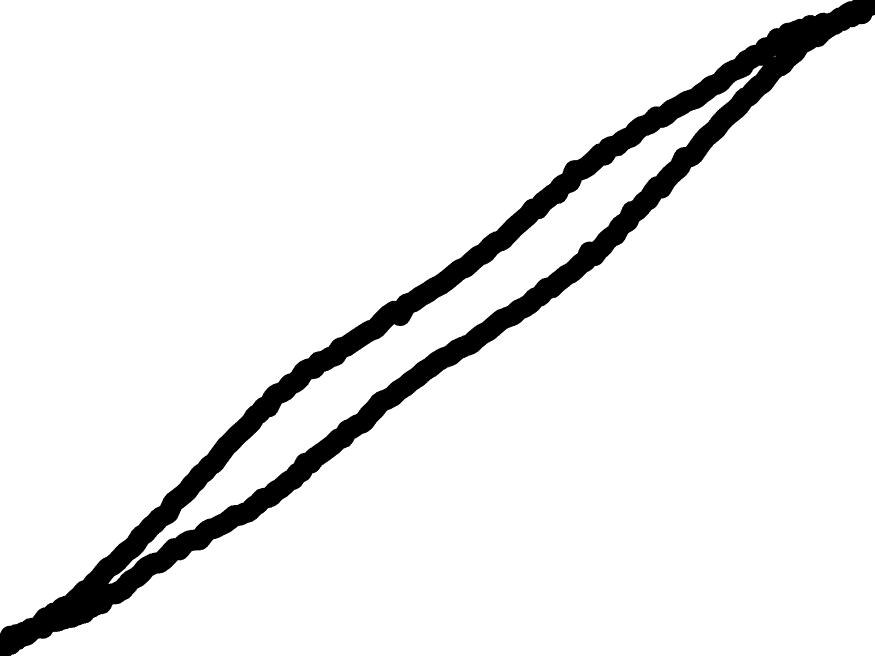

Supplement: S1 Data — (ZIP) [file pone.0312954.s001.zip › S1 data/1.V-I trajectories/0/1 highcool_1057/281_0.jpg]
