# Supplementary material for: Two-stage Non-Intrusive Load Monitoring method for multi-state loads
Source: PLoS One. 2025 Jan 8;20(1):e0312954. doi: 10.1371/journal.pone.0312954 (PMC11709270; doi:10.1371/journal.pone.0312954)
Supplement: S1 Appendix — (DOCX) [file pone.0312954.s002.docx]

S1 Appendix

The brief expression and the summary tables of different models have been presented.

1) VGG16

The original VGG16 model cannot be directly applied to the classification task of the NILM. In this paper, the VGG16 model is tailored through parameter adjustments to meet the demands of load identification. After passing through 13 convolutional layers and 4 pooling layers, the input images are transformed and then fed into fully connected layers. Among them, convolutional layers for computing image features and fully connected layers alternate, forming five distinct segments separated by pooling layers. The model incorporates three fully connected layers dedicated to classification, with the first two layers each containing 4096 neurons. The final fully connected layer generates a vector with a number of elements equal to the 11 electrical appliance categories to be identified. To accelerate the model training process and prevent overfitting, dropout layers are inserted after each fully connected layer, with a dropout rate of 0.5. The structure of the VGG16 model used in this paper is outlined in the table below.

Table 1. Kernel Functions of VGG16 Model Structure

| Type | Kernel Functions | Activation Function |
| --- | --- | --- |
| input layer |  |  |
| convolutional layer | 3*3（64） | Relu |
| convolutional layer | 3*3（64） | Relu |
| max pooling layer | 2*2 |  |
| convolutional layer | 3*3（64） | Relu |
| convolutional layer | 3*3（64） | Relu |
| max pooling layer | 2*2 |  |
| convolutional layer | 3*3（64） | Relu |
| convolutional layer | 3*3（64） | Relu |
| convolutional layer | 3*3（64） | Relu |
| max pooling layer | 2*2 |  |
| convolutional layer | 3*3（128） | Relu |
| convolutional layer | 3*3（128） | Relu |
| convolutional layer | 3*3（128） | Relu |
| max pooling layer | 2*2 |  |
| convolutional layer | 3*3（128） | Relu |
| convolutional layer | 3*3（128） | Relu |
| convolutional layer | 3*3（128） | Relu |
| fully connected layer | 4096*1 | Relu |
| dropout layer(0.5) |  |  |
| fully connected layer | 4096*1 | Relu |
| dropout layer (0.5) |  |  |
| fully connected layer | 11*1 | SoftMax |
| output layer | 11*1 | SoftMax |

2) Resnet18

The structure of Resnet18 model used in this paper is shown in Fig 2. The original Resnet18 model cannot be directly applied to the classification of the Plaid dataset. Therefore, the model is modified by improving its parameters, which is adapted to the requirements of load identification. The input image passes through a convolutional layer and enters 8 Resnet blocks, which consisting of two convolutional layers. Finally, a fully connected layer is used for classification, and the number of vectors generated is equal to the number of appliance categories to be recognized 11. The model is trained for 800 epochs with a learning rate of 0.0001.

Fig 1. Resnet18 model structure.

3) K-Means

In paper, the value of k is chosen as 11 (representing 11 cluster centers), and Euclidean distance is used as the method to measure the distance between data points.

Table 2. Parameters of K-Means

| Type | Parameters |
| --- | --- |
| Number of clustering | K=11 |
| Method of calculating distance | Euclidean distance |
| Calculation formula | $\rho=\sqrt{\text{（}x_{2}-x_{1})^{2}+(y_{2}-y_{1})^{2}}$ |

4) SVM

To identify 14 working states, 14 classifiers are required to be built, aiming to improve the training speed and recognition accuracy of the algorithm, reduce training time, and enhance recognition efficiency. The SVM classifiers are trained using the One-versus-all (OVA) method to construct 14 classifiers, with a linear kernel function, a C-value of 1, and a random state of 0.

Table 3. Parameters of SVM

| Type | Parameters |
| --- | --- |
| Construction Method | OVA |
| Number Of Classifiers | 14 |
| Function | Linear Kernel Function |
| C | 1 |
| Random State | 0 |
